# Spatial analysis of 10-year predicted risk and incident atherosclerotic cardiovascular disease: the CoLaus cohort

**DOI:** 10.1038/s41598-024-54900-5

**Published:** 2024-02-27

**Authors:** Guillaume Jordan, David Ridder, Stephane Joost, Peter Vollenweider, Martin Preisig, Pedro Marques-Vidal, Idris Guessous, Julien Vaucher

**Affiliations:** 1 Department of Medicine, Division of Internal Medicine, Lausanne University Hospital and University of Lausanne, Rue du Bugnon 46, 1011 Lausanne, Switzerland; 2 Department of Primary Care Medicine, Division of Primary Care Medicine, Geneva University Hospitals, Geneva, Switzerland; 3 Laboratory for Biological Geochemistry (LGB), Group of Geospatial Molecular Epidemiology (GEOME), Institute of Environmental Engineering, Ecole Polytechnique Fédérale de Lausanne (EPFL), Lausanne, Switzerland; 4 CEPP, Department of Psychiatry, Lausanne University Hospital and University of Lausanne, Prilly, Switzerland; 5 Department of Medicine and Specialties, Service of Internal Medicine, Fribourg Hospital and University of Fribourg, Fribourg, Switzerland; 6 Geographic Information Research and Analysis in Population Health (GIRAPH), Geneva, Switzerland; 7 Faculty of Medicine, University of Geneva, Geneva, Switzerland

**Keywords:** Risk factors, Cardiovascular diseases, Predictive markers, Preventive medicine, Epidemiology, Environmental impact

## Abstract

Whether cardiovascular risk scores geographically aggregate and inform on spatial development of atherosclerotic cardiovascular diseases (ASCVD) remains unknown. Our aim is to determine the spatial distribution of 10-year predicted cardiovascular risk and ASCVD, and to compare the overlap of the resulting spatial distributions. Using prospective data from the CoLaus|PsyCoLaus cohort study (2003–2021) we computed SCORE2 in participants free from ASCVD. Geographical distributions of predicted risk and events were determined using the Gi* Getis-Ord autocorrelation statistic. 6203 individuals (54% women, mean age 52.5 ± SD 10.7, ASCVD incidence rate 5.7%) were included. We identified clusters of high versus low predicted risk (4%, 6%, respectively) and ASCVD (5%, 5% respectively) at baseline. They persisted at follow-up. Overlap of SCORE2 and ASCVD clusters was marginal. Body-mass index and alcohol consumption explained most of the predicted risk distribution. For ASCVD, high clusters persisted or were reinforced after multivariate adjustment, while low incidence clusters were reduced, multifactorial determinants. Incidence rate of ASCVD was 2.5% higher (IC 95%, 1.4–3.7) in clusters of higher incidence of ASCVD. To develop up-to-date, geographically targeted prevention strategies, there is a need to study novel geographically risk factors affecting ASCVD and to update commonly used prediction models for a population approach.

## Introduction

Cardiovascular disease (CVD) is a leading cause of death and represents an important societal, and economic burden^1^. By shifting towards non-fatal outcomes, it has become a major chronic disease^2–6^.

Given that incidence of CVD varies geographically, it is crucial to understand the distribution of the disease to develop cost-effective and priority-based prevention strategies^7–9^. Spatial studies, mainly cross-sectional, have already shown the existence of CVD clusters and cardiovascular risk factors^10–14^.

Cluster of CVD have been shown to change overtime, underlining the opportunity that prediction models could offer as a precision public health tool to assess cardiovascular risk factors dynamically to design up-to-date public health strategies^11^. So far, a few studies have focused on the spatial distribution of predicted cardiovascular risk^15,16^. In 2017, Dalton, al. found that the Pooled Cohort Equations Risk Model accounted only modestly for the spatial variability in CVD event rates in north-eastern Ohio^16^. Whether predicted cardiovascular risk and atherosclerotic CVD (ASCVD) are congruent in a spatial manner remains unknown.

Therefore, using prospective data of a population-based study, the study first sought to determine the spatial distribution of 10-year predicted cardiovascular risk and ASCVD, and to compare the overlap of the resulting spatial distributions.

## Methods

### Study design and participants

This study used prospective data from the existing CoLaus|PsyCoLaus study (www.colaus-psycolaus.ch), a Swiss population-based cohort in the city of Lausanne^7^. Between 2003 and 2006, 6733 subjects (age range 35–75 years, 54% women) were randomly recruited. A complete list of Lausanne inhabitants was provided by the population register of the city and was used to sample the participants to the study. A simple, non-stratified random selection of 19,830 subjects, corresponding to 35% of the source population, was drawn using STATA v9.1 software (Stata Corp, College Station, USA), and a letter inviting the addressee to participate in the study was sent to these individuals. The following inclusion criteria were applied: (a) written informed consent; (b) age 35–75 years and (c) Caucasian origin (91.9% of the Lausanne population). Caucasian origin was defined as having both parents and grandparents born in a restricted list of countries (available from the authors). No other exclusion criteria were applied.

The assessment process, the questionnaire, clinical and biological data collection, the definition and adjudication of cardiovascular can be found in the Supplementary material. Further details about this cohort study can be found in the original article^7^.

Periodic resurveys of the whole cohort were conducted over an 18-year follow-up. Patients or the public were not involved in the design and conduct of the study. As this study consists of a secondary data analysis, sample size was already defined.

### Ethics declaration

The institutional Ethics Committee of the University of Lausanne, which later became the Ethics Commission of Canton Vaud (www.cer-vd.ch) approved the CoLaus|PsyCoLaus study (www.cer-vd.ch; project number PB_2018-00038, reference 239/09). All participants provided written informed consent. This study conforms to the Declaration of Helsinki.

### Inclusion criteria

For the present study, we have included participants of the CoLaus|PsyCoLaus study without pre-existing ASCVD and with geocoding corresponding to the urban districts of Lausanne. Participants with missing data for risk calculation were excluded. Participants developing ASCVD before the onset of the follow-up were excluded from analyses corresponding to that period.

### Geocoding

Participants were georeferenced at their postal address through the joint use of the Swiss Confederation's geocoding application, the GEOADMIN API (https://api3.geo.admin.ch/) and the Register of Buildings, and Housing REGBL (https://www.housing-stat.ch/fr/index.html). Geocoding was repeated at each follow-up. Urban districts only were considered because they were dense enough to allow for spatial analyses.

### Risk prediction models

Our primary endpoint, 10-year predicted risk was computed at the first two periods of the four follow-ups that were conducted in the study, baseline (2003–2006), follow-up 1 (2009–2012), as to be able to compare its distribution with that of the 10-year incident ASCVD. We computed the Systematic Coronary Risk Evaluation Score 2 (SCORE2) and Systematic Coronary Risk Evaluation Score 2-older persons (SCORE2-OP) for participants of age 35–69, and ≥ 70 years, respectively^5,17^. Both were calibrated for a low-risk region.

SCORE2 and SCORE2-OP are sex and competing-risk adjusted predictions models, recalibrated to four defined European risk regions, to evaluate 10-year cardiovascular risk defined as non-fatal myocardial infarction and stroke in apparent healthy individuals. They have been developed by the SCORE2/SCORE2-OP working group and ESC Cardiovascular risk collaboration. The models included the following predictors: age, current smoking, history of diabetes mellitus, systolic blood pressure, and total- and HDL-cholesterol.

In this cohort, SCORE2 and SCORE2-OP were computed using the available algorithms offered by the SCORE2 working group and ESC Cardiovascular risk collaboration using the software Stata (StataCorp. 2021. Stata Statistical Software: Release 17. College Station, TX: StataCorp LLC)^5,17^.

Participants suffering from diabetes, chronic renal kidney disease and possible familial hypercholesterolemia (Low-density lipoprotein cholesterol (LDL-C) > 4.9 mmol/L or Total cholesterol > 7.5 mmol/L) were reclassified according to their corresponding categories of cardiovascular risk as defined by the 2021 ESC Guidelines on cardiovascular disease prevention in clinical practice^3,18^.

Reclassified participants were assigned a predicted risk equal to the inferior threshold of their corresponding category of risk.

### Incident atherosclerotic cardiovascular diseases

Ten-year incident ASCVD, as a secondary endpoint, was considered 10 years after baseline visit (2003–2006) and follow-up 1 (2009–2012) for each participant. ASCVD was defined as the presence of either an episode of: (a) acute coronary syndromes (ACS) (acute myocardial infarction (AMI) or unstable angina); (b) sudden cardiac death; (c) symptomatic coronary artery disease (CAD) with greater than 50% stenosis (treated by percutaneous coronary intervention or coronary artery bypass graft); and (d) fatal and non-fatal ischaemic stroke (including transient ischaemic attack).

Medical records of participants who declared an incident ASCVD and/or ASCVD-related procedure were prospectively collected, as well as information on cause of death. Outcomes were independently adjudicated by trained specialists (i.e., cardiologists, neurologists, and internists). The complete procedure has been previously reported^19^.

### Statistical analysis

The geographical distribution of predicted risk and incident ASCVD was assessed using spatial autocorrelation measures, that enable the study of smaller areas, not delimited by administrative boundaries^10,20–22^. Those methods identify clusters of areas or individuals, in which a variable of interest is more closely related to its surrounding than to regions that are more distant^23–25^.

Analyses were conducted using incremental fixed distance bands (200 m, 400 m, 600 m, 800 m, 1000 m, and 1200 m) as regions of interest. Additionally, assessments were repeated using a k-nearest neighbours weights method.

Autocorrelation was also stratified by sex assigned at birth, self-reported, and measured on the mean squared error between predicted probabilities or risk and observed values (ASCVD), equivalent to a Brier score (Supplementary material), to identify areas where prediction models lack performance.

To test for global spatial autocorrelation of predicted risk, overall tendency of clustering, the Global Moran’s I statistic was used. Because it tends to average local variations, this method does not rule out local clusters. To test for local spatial autocorrelation, to find such clusters, Getis-Ord Gi* statistic was used for continuous and binary variables. A pseudo-*p* value > 0.05 based on 999 random permutations was considered.

We conducted exploratory analyses to investigate whether covariates may be associated with clusters. Concisely, spatial autocorrelation was measured before and after adjustment, using residuals of univariable and incremental multivariable regressions models^12^. A reduction of the clustering would indicate a positive dependence upon the covariates. We used both traditional OLS, logistic and geographically weighted models (Multi-scale Geographically Weighted Regression (MGWR), Geographically Weighted Regression (GWR)) that allowed relationships to vary across the study area^26^.

Such models result in multiple coefficients of regression across the area. The coefficient of regression are represented on maps, called parameter estimates surfaces, that can be found in the Supplementary material. Models fit metrics were R-squared, percent deviance explained and AICc. Multicollinearity was investigated using variance inflation factor (VIF) and local condition number (CN) (supplementary figure S1-2).

Regarding predicted risk, adjustment analyses were conducted on predicted risk in which age was fixed using the population mean age as a constant, to focus on geographical associations that were independent of age distribution, which is not a modifiable risk factor.

Covariates were selected after conduction of a scoping review on cardiovascular risk factors and according to data availability. Such variables were weekly alcohol consumption and body mass index (BMI) in model one, run on predicted risk. Low education status, Townsend deprivation index per hectare, Swiss origin and living in a couple were added in model two (socio-economic status variables) and, additionally, existence of anxiety disorder, and major depressive disorder in model three (psychological risk factors). Model four consisted in the same variables as model three with the addition of a polygenic risk score, a Mediterranean diet score and daily minutes of moderate intensity sport activity at follow-up 1. Model four included variables that were only available at one period, the first follow-up.

Models run on ASCVD and on the Brier score included the same variables used in model 4 previously described, with the addition of age, existence of an antihypertensive or lipid modifying drug, presence of either diabetes, chronic renal kidney disease, or possible familial hypercholesterolemia (labelled as comorbidity), and predicted risk with age fixed in one additional model ran on ASCVD. Covariates distribution in our sample can be found in supplementary table S1.

Additionally, Wilcoxon, non-parametric, and Chi-square tests for binary and numeric variables were conducted to calculate risk and incidence rate differences between high or low clusters and the rest of the population (supplementary table S2).

Results are presented using mean and standard deviation (SD) for normal variables, median and interquartile range (IQR) for skewed variables, parameter estimates, model fits metrics and dot maps. Overall performance of risk prediction models was evaluated using ROC curves with 95% IC, calibration plots and Brier scores.

Spatial analyses results are presented considering a 600 m bandwidth, the smallest fixed band without isolates which also displayed well-circumscribed clusters in comparison to smaller perimeters. Statistical analysis was conducted using Stata (StataCorp. 2021. Stata Statistical Software: Release 17. College Station, TX: StataCorp LLC), GeoDa, 1.20 (Spatial Analysis Laboratory, University of Illinois, USA9), and MGWR (School of geographical sciences and urban planning, Arizona State University, USA). For further details, refer to Supplementary methods.

### Missing data

Participants without any data for risk computation or geocoding (154, 2.4%) were excluded from analyses. To understand effect of loss of participants in between follow-ups on geographical distribution, we restricted analysis on participants present a both timelines. To account for loss of participants due to reclassification, we conducted analyses on non-reclassified predicted risk (Supplementary results).

Characteristics of excluded and included participants were described to better understand effect of missing data on sample representation. Further details are presented within the Supplementary material.

### Sensitivity analyses

All sensitivity analyses were conducted based on a 600 m bandwidth. We conducted analyses only on individuals without diabetes, chronic kidney disease or familial dyslipidaemia to assess the performance of the model in an apparent healthy population.

Second, to allow for the use of the SCORE2 model only, we repeated analyses excluding participants over 70 years. Autocorrelation on difference of predicted risk between periods, and on ESC risk categories as binary variables was measured (data not shown).

Finally, to assess the impact of loss to follow-up and house moving on geographical distribution of clusters, analyses for each outcome variable of each period were conducted using iteratively geographical coordinates at baseline and at follow-up. Additionally, the latter analyses were restricted to the subset of individuals who did not move during the study to differentiate a relation mediated by house moving from one mediated by attrition. Additional sensitivity analyses (supplementary figure S3-5) are presented within the Supplementary material.

## Results

### Study population and endpoints

Of the initial 6733 participants, 6203 (92%, 54% women, mean age 52.5 ± SD 10.7, supplementary figure S6) at baseline and 4206 (83%, 56% women, mean age 58 ± SD 10, supplementary figure S6) at first follow-up were included and analyzed, with a median follow-up of ten (interquartile range (IQR), 6–10) and nine (IQR 5–9) years, respectively. Characteristics are shown in Table 1.

**Table 1:** Participants’ characteristics at baseline (2003–2006) and follow-up 1 (2009–2012).

	Baseline (2003–2006)	Follow-up 1 (2009–2012)
N	6203	4206
Follow-up duration [years], median (IQR)	10 (6–10)	9 (5–9)
Male sex, n (%)	2861 (46)	1867 (44)
Age [years], mean ± SD	52 ± 11	58 ± 10
Current smoker, n (%)	1670 (27)	919 (22)
Blood pressure [mmHg], mean ± SD		
Systolic	128 ± 18	126 ± 18
Diastolic	79 ± 11	78 ± 11
Total cholestérol [mmol/L], mean ± SD	5.6 ± 1	5.7 ± 1
Hdl-cholestérol [mmol/L], mean ± SD	1.6 ± 0.4	1.6 ± 0.5
Ldl-cholestérol [mmol/L], mean ± SD	3.3 ± 1	3.5 ± 1
Hypertension, n (%)	2220 (36)	1666 (40)
Diabetes, n (%)	351 (6)	416 (10)
Chronic kidney disease, n (%)	262 (4)	266 (6)
Lipid modifying agents, n (%)	678 (11)	693 (16)
Antihypertensive agents, n (%)	1107 (18)	1048 (25)

Description with mean and SD of variables showing a normal distribution and using median, and interquartile range for skewed variables. SD^a^, standard deviation; IQR, interquartile range; n, number.

Included participants were healthier than excluded ones (supplementary figure S7, supplementary table S3). Distribution in characteristics, zip codes and covariates were similar between follow-ups (Table 1).

Median predicted risk by SCORE2 and SCORE2-OP combined was 2.9% [1.4–5.4] at baseline and 4% [2–6.9] at first follow-up (supplementary table S4). Ten-year incident ASCVD was 5.7% and 6.7% at baseline and first follow-up, respectively (supplementary table S4).

### Geographical distribution of 10-year predicted risk

**Figure 1:**
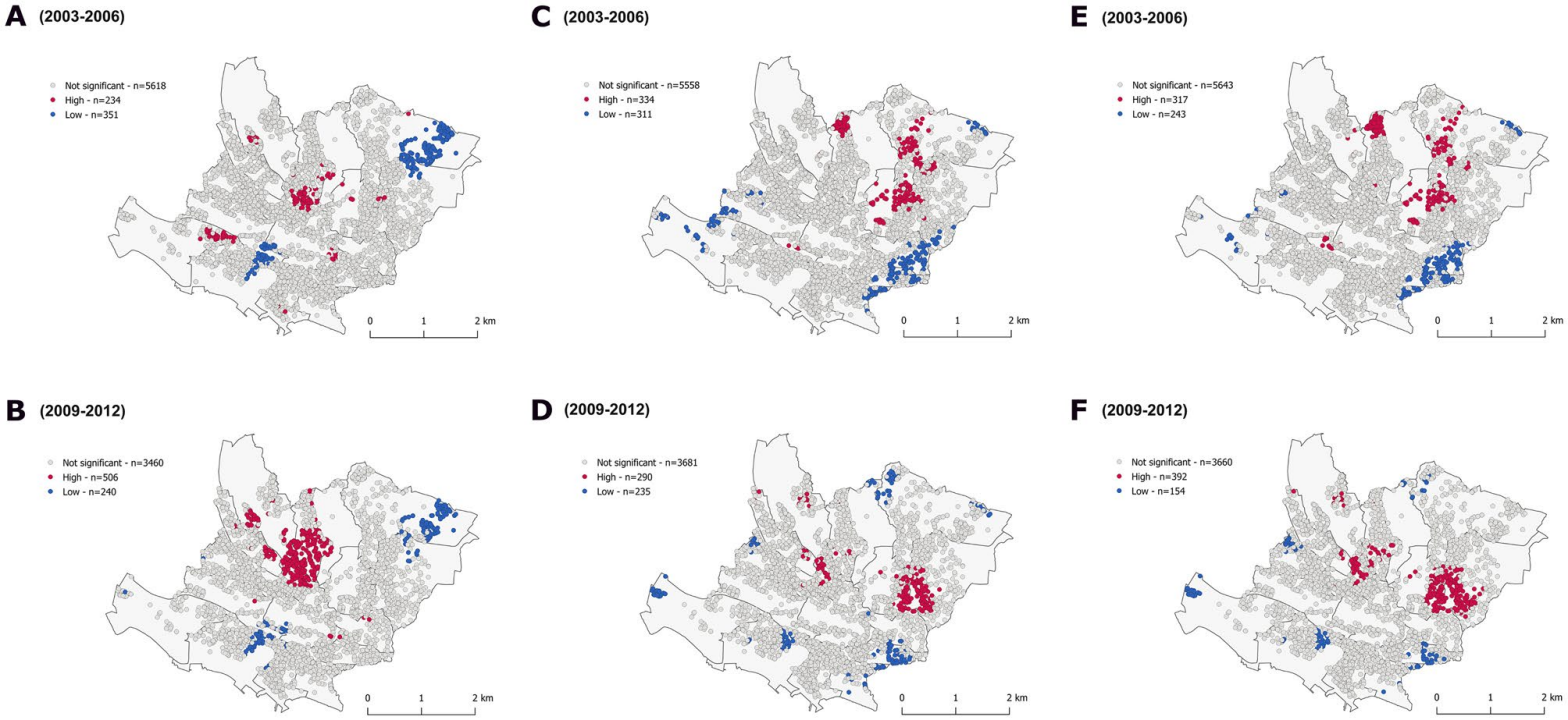
Clusters of predicted risk (**A**,**B**), 10-year observed cardiovascular diseases (**C**,**D**) and mean squared errors of prediction (**E**,**F**). Getis-Ord Gi* statistic for the individuals geo-referenced at their postal address. White dots correspond to individual with values that do not exhibit spatial dependence. Red/Blue dots indicate individuals with a positive/negative Z score considering a *p* value < 0.05 (α = 0.05). This means that high or low values clusters, respectively, within a spatial lag of 600 m, are found closer together than expected if the underlying spatial process was random.

First, we assessed the geographical distribution of SCORE2. While there was no global autocorrelation (supplementary table S5), we found local spatial clusters of high (4%, pseudo-*p* < 0.05 at baseline, < 0.001 at follow-up) and low (6%, pseudo-*p* < 0.001 at baseline and follow-up) predicted risk (Fig. 1). Computed risks were 3% [IQR 1.5–5.7] and 2.7% [IQR 1.3–5.1] at baseline, in high and low clusters, respectively, and 4.1% [IQR 2.1–7.2], 3.9% [IQR 2–6.6] at follow-up (supplementary table S6).

Spatial distribution was relatively consistent between bandwidths, types of weighting schemes (*data not shown*) and over time (Fig. 1). Sensitivity analyses, shown within the Supplementary material, confirmed the robustness of our results, as we found similar results within an under 70-year population, when using SCORE2 only (supplementary figure S8) or when restricting analyses to an apparent healthy population (supplementary figure S9).

Regarding the temporal changes in clusters of predicted risk, we mainly see a reinforcement of the central area on the map. These results could neither be explained by loss of participants nor house moving as shown on supplementary figure S10. The significance of this change was confirmed by further spatial auto-correlation analyses on the distribution of the difference of risk in between the two follow-ups (*data not shown)*.

### Geographical distribution of 10-year incident atherosclerotic cardiovascular diseases

Then, we assessed the distribution of 10-year incident atherosclerotic cardiovascular diseases. We found local clusters of high (5%, pseudo-*p* < 0.001, at baseline and follow-up) and low (5%, pseudo-*p* < 0.01 at baseline, < 0.001 at follow-up) incident ASCVD (Fig. 1). Results were robust according to most of our sensitivity analyses (Supplementary material).

Between baseline and follow-up, a small cluster appeared on the centre of the map and a north cluster disappeared, while the majority eastern clusters persisted. The reinforcement was not related to house moving according to our sensitivity analyses (supplementary figure S10). We were able to recreate similar patterns at baseline by excluding participants that were lost to follow-up, suggesting the changes could be related to an attrition or exclusion mediated effect (supplementary figure S10).

Cumulative incidence rates of events were 7% (n = 214) and 4.4% (n = 127) at baseline, within high and low clusters, respectively, and 7.7% (n = 166), and 5.2% (n = 123) at follow-up (supplementary table S6).

Incidence rate differences over 10 years between clusters of high incidences of ASCVD and the rest of the sample were 2.5% (95% confidence interval (IC 95%), 1.4, 3.7) at baseline and 2.1% (IC 95%, 0.5, 5.8) at follow-up. Incidence rate differences over 10 years between clusters of low incidences of ASCVD and the rest of the sample were − 2.4% (IC 95%, − 1.3, − 3.5) at baseline and − 3.3% (IC 95%, − 1.8, − 5) at follow-up (supplementary table S2).

### Adjustment

**Figure 2:**
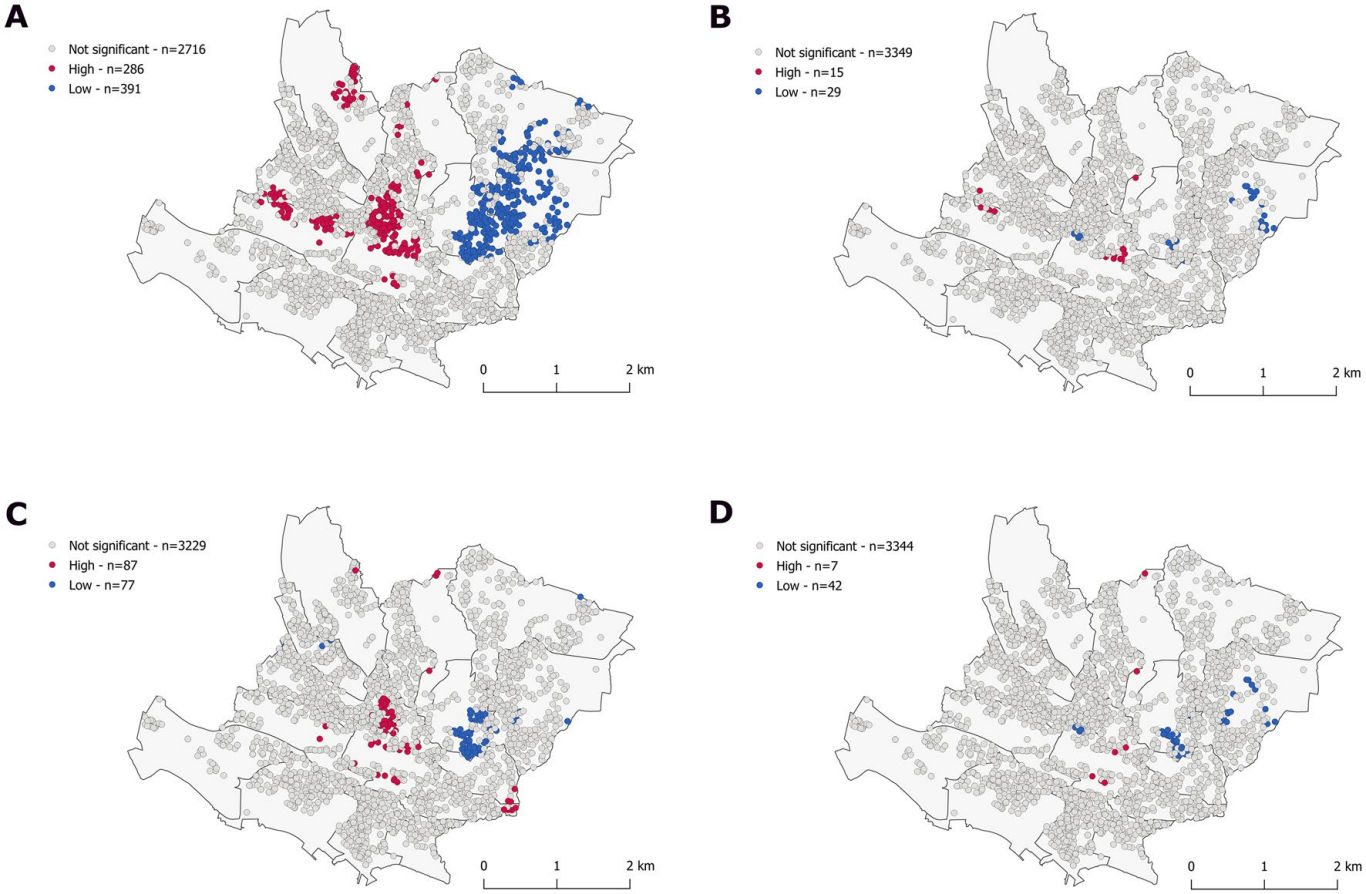
Clusters of predicted risk, with age fixed, unadjusted (**A**), adjusted for alcohol consumption (**B**), body mass index (**C**) and multivariable adjusted at baseline (2003–2006). Getis-Ord Gi* statistic for the individuals geo-referenced at their postal address. White dots correspond to individual with values that do not exhibit spatial dependence. Red/Blue dots indicate individuals with a positive/negative Z score considering a *p* value < 0.05 (α = 0.05). This means that high or low values clusters, respectively, within a spatial lag of 600 m, are found closer together than expected if the underlying spatial process was random. The multivariable model (MGWR) included body mass index, alcohol consumption, living in couple, low education status, Townsend index, Swiss origin, anxiety disorder and major depressive disorder, a polygenic risk score and at follow-up 1 (2009–2012) a Mediterranean diet score, and daily moderate intensity physical activity.

**Figure 3:**
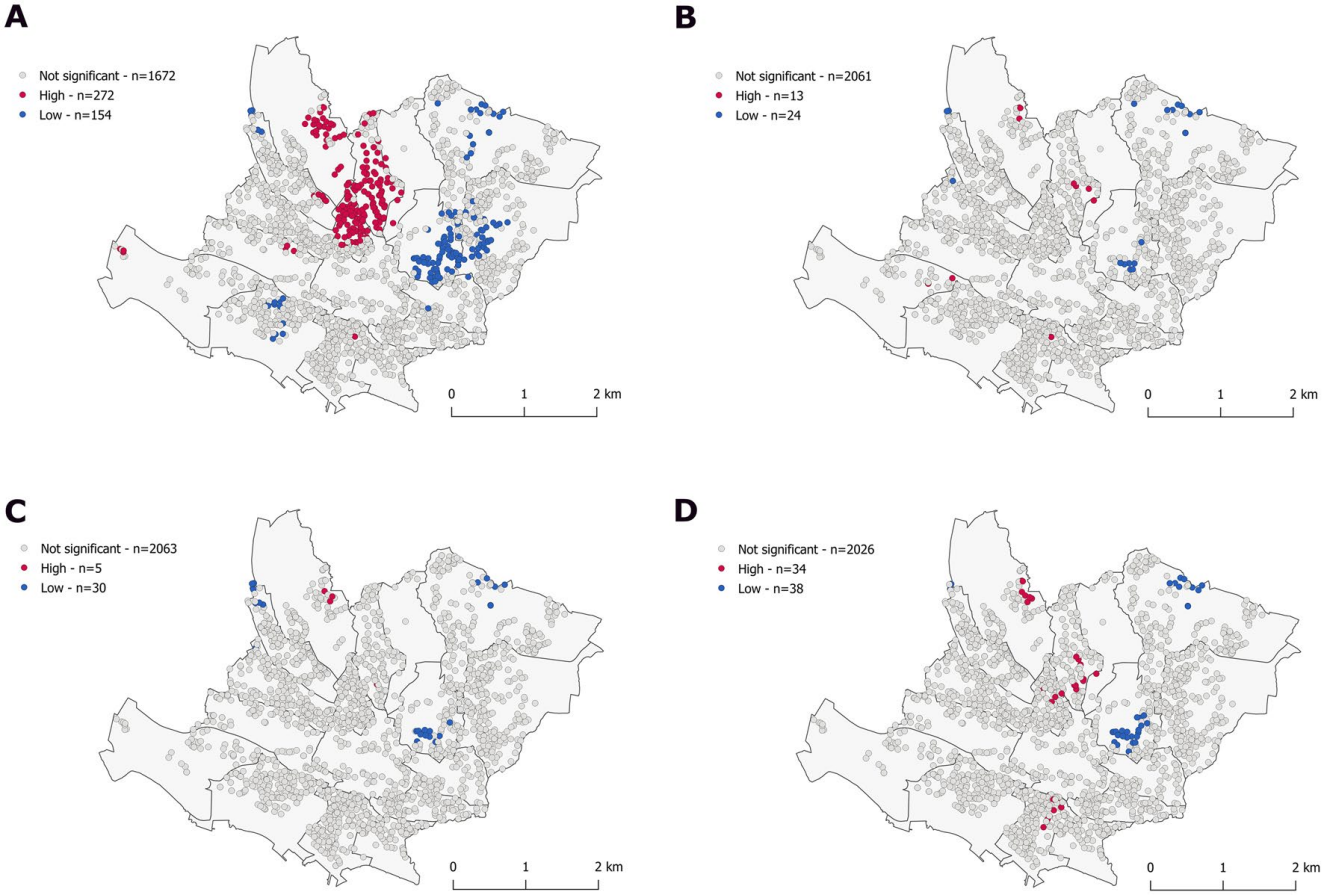
Clusters of predicted risk, with age fixed, unadjusted (**A**), adjusted for alcohol (**B**), body mass index (**C**) and multivariable adjusted at follow-up 1 (2009–2012). Getis-Ord Gi* statistic for the individuals geo-referenced at their postal address. White dots correspond to individual with values that do not exhibit spatial dependence. Red/Blue dots indicate individuals with a positive/negative Z score considering a *p* value < 0.05 (α = 0.05). This means that high or low values clusters, respectively, within a spatial lag of 600 m, are found closer together than expected if the underlying spatial process was random. The multivariable model (MGWR) included body mass index, alcohol consumption, living in couple, low education status, Townsend index, Swiss origin, anxiety disorder and major depressive disorder, a polygenic risk score and at follow-up 1 (2009–2012) a Mediterranean diet score, and daily moderate intensity physical activity.

Next, we conducted exploratory analyses to to investigate whether covariates may be associated with clusters. Regarding predicted risk, the analyses showed that clusters were greatly attenuated at baseline and follow-up after adjustment (Figs. 2, 3). The MGWR model displayed the best model fit metrics (supplementary table S7). Alcohol consumption and BMI were most closely associated with the clustering tendency (Figs. 2, 3; supplementary figure S11-13, 1, 2, supplementary table S8-9).

In our linear multivariate regression models, BMI, alcohol consumption, low education, living in couple and the Townsend index were all positively associated with cardiovascular risk (*p* value < 0.001). Addition of the latter socio-economic variables to a model containing BMI and alcohol did not alter the magnitude of the association of BMI and alcohol with cardiovascular risk. Conversely, adding BMI and alcohol to models comprising low education, living in couple and the Townsend index resulted in an approximately 50% reduction of the magnitude in the corresponding beta-coefficients.

**Figure 4:**
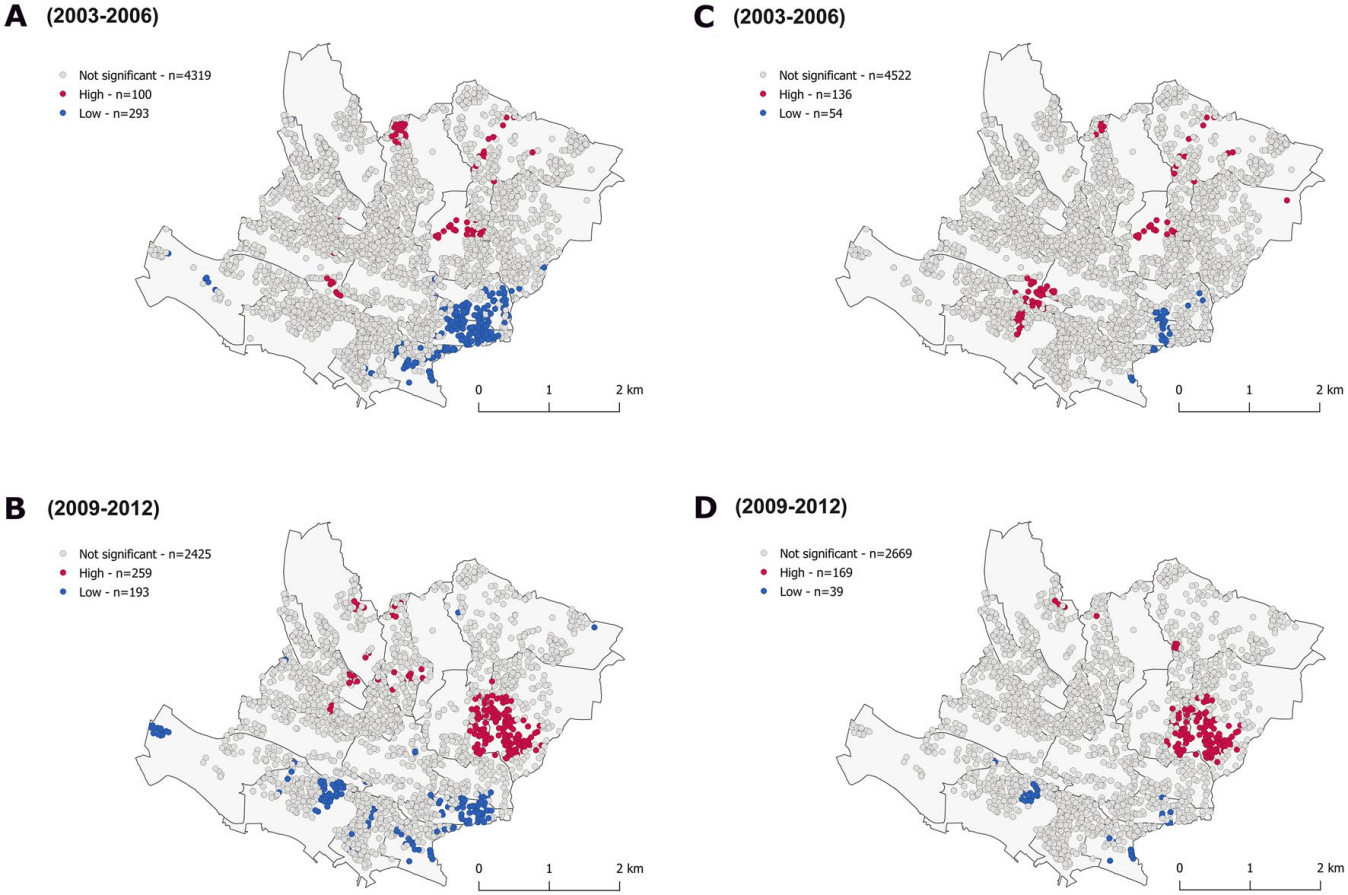
Clusters of 10-year observed cardiovascular diseases unadjusted (**A**,**B**) and multivariable adjusted (**C**,**D**). Getis-Ord Gi* statistic for the individuals geo-referenced at their postal address. White dots correspond to individual with values that do not exhibit spatial dependence. Red/Blue dots indicate individuals with a positive/negative Z score considering a *p* value < 0.05 (α = 0.05). This means that high or low values clusters, respectively, within a spatial lag of 600 m, are found closer together than expected if the underlying spatial process was random. The multivariable model (GWR) included age, comorbidity, lipid modifying and antihypertensive drug, body mass index, alcohol consumption, living in couple, low education status, Townsend index, Swiss origin, anxiety and major depressive disorder, the predicted risk with age fixed and at follow-up 1 a Mediterranean diet score, and daily moderate intensity activity. Additional models accounting for a polygenic risk score, with similar results, are shown within the supplementary figure S14, 18.

Regarding incident ASCVD, attenuation was found for low clusters with similar results between GWR and OLS (Fig. 4; supplementary figure S14-20, supplementary table S10). High risk clusters persisted after adjustment, some were even reinforced (Fig. 4). Similar results were found with applying MGWR to the mean squared error between predicted probabilities and observed values (Fig. 4, supplementary figure S21, 22, supplementary table S11).

The age, the polygenic risk score and the predicted risk, displaying a non-hazardous association with ASCVD (supplementary table S12), contributed only modestly to the attenuation of low clusters in univariable models, suggesting the adjustment was multifactorial (supplementary figure S16, 18, 23). The polygenic risk score correlated to the mean squared error between predicted risk and events (β 0.15 at baseline, 0.09 at follow-up, *p* value < 0.05, supplementary table S13).

### Overlap of distribution between predicted risk and incident atherosclerotic cardiovascular diseases

Finally, we assessed the overlap of distribution between predicted risk and incident atherosclerotic cardiovascular diseases. There was no overlap at baseline and minor overlap at follow-up between high and low clusters of predicted risk and incident ASCVD, independently of statistical method chosen, before and after adjustment (Figs. 1, 2, 3, 4). Results were identical when mapping the mean squared error between predicted probabilities and observed values (Fig. 1). Inter-cluster differences were more marked for ASCVD than predicted risk.

The overlap slightly improved at follow-up in the centre of the map, with the appearance of a small cluster of ASCVD in the centre of the map. This cluster did not disappear after adjustment of ASCVD for predicted risk (supplementary figure S22). This dynamic pattern could be related to attrition.

Restricting analyses to healthy individuals improved overlap, mainly at follow-up (supplementary figure S9). However, discrepancy persisted after adjustment of ASCVD for comorbidities (supplementary figure S17). Clusters of mean squared errors between predicted risk and 10-year incident events stayed alike.

## Discussion

As hypothesized, we identified clusters of higher and lower predicted risk and incidence of ASCVD, respectively, which, surprisingly, did not overlap. Unlike for predicted risk clusters, traditional, behavioural, socio-psychological, and genetic risk factors did not explain ASCVD distribution.

### Geographical distribution of 10-year predicted risk

Our findings highlighted a tendency of local clustering of predicted risk, in line with Bagheri, al. using the Framingham risk equation in a cross-sectional sample of general physician patients, northwest Adelaide , South Australia, 2012^15^. In comparison, our results showed a narrower range of 10-year predicted risk.

At follow-up, we highlighted a reinforcement in the central area of the map, which was confirmed by sensitivity analyses on the difference in risk in between periods. It should be noted that the concerned central area consists of a region with a known higher rate of smoker and lower levels of HDL-cholesterol in average, as supported by the results of Juan R. Vallarta-Robledo et al.^27^.

### Geographical distribution of 10-year incident atherosclerotic cardiovascular diseases

Our results demonstrated a tendency of local clustering of ASCVD. Such clusters have already been identified in a few studies^10,11,16,28,29^. While most of clusters in our study were consistent, some changed over time in agreement with Rajabi et al.^11^, confirming the relevance of having a dynamic approach towards risk assessment.

There was a 2.5% variability in incidence rate of ASCVD over 10 years between clusters of higher or lower incidence of ASCVD and the rest of the population, which is clinically meaningful considering an average 10-year incident rate of approximately 6% in the whole population.

While many clusters stayed alike, we observed shifts in patterns in between periods. Most of changes could be explained by the loss of participants. Our main hypothesis is that some participants who developed an ASCVD were excluded from the analyses in follow-ups based on selection criteria. These ‘missing’ participants, could then have been responsible for the shift in patterns. It is interesting to note the emergence of a central cluster of ASCVD at follow-up 1, overlapping the clusters of high predicted risk. Our interpretation is that factors comprised in SCORE2 do drive ASCVD but fail to capture the total risk over the time and in a given area. This should stem from the fact that total risk is composed of other known and unknown non-observed factors.

### Adjustment

In our exploratory analyses, predicted risk clusters were mainly associated with BMI and alcohol consumption. The results are supported by the fact that the geographical distribution of regression coefficients for those variables persisted after adjustment and that the relationship between alcohol consumption, BMI and ASCVD were found in a small perimeter.

Regarding incident cardiovascular events, we found no association between all studied covariates and high-risk ASCVD clusters. In low-risk clusters, attenuation was multifactorial. In the absence of association with behavioral, psychological, socio-economic status variables and traditional cardiovascular risk factors, our hypothesis is that environmental variables, such as pollution, landscape architecture and noise, are highly likely to be the major driver of distribution of cardiovascular risk in this sample. There is a need to deepen our understanding of these novel risk factors. It should be pointed that this result might suffer from an ecological fallacy and might only apply to a similar environment.

Other studies have found a spatial association between CVD and socio-economic characteristics such as education and material deprivation^10^. The discrepancy with our results could possibly be related to a lack of variability in social economic factors in Switzerland, in fact the difference in Townsend scores between the most deprived and wealthiest areas was very narrow in our study.

Our linear multivariate models confirmed a non-spatial association between socio-economic status and cardiovascular risk and suggested it contributes to risk through BMI and alcohol consumption but not in the opposite direction.

### Overlap of distribution between predicted risk and incident atherosclerotic cardiovascular diseases

Our results showed marginal overlap between predicted risk using SCORE2 and ASCVD geographical distributions.

In a previous study, Dalton, al. found that the Pooled Cohort Equations Risk Model accounted for ten percent of the spatial variability in ASCVD event rates, in a cohort in the United States^16^. Neighbourhood deprivation accounted for 32% of the variability, which was not the case in our sample. 60% of the variability was unexplored in their study.

Another study by Van Rheenen, al. highlighted a marginal overlap between clusters of stroke risk factors and of ischemic stroke^30^. Quality of care associated with having a reported risk factor was hypothesized as the associated factor, however this data lacked in the study. In our study, the discrepancy was robust to adjustment for treatment of absolute risk by antihypertensive and lipid modifying agents.

Inter-cluster differences were more marked for ASCVD than predicted risk, suggesting the map of predicted risk was more homogeneous and that SCORE2 could have underperformed due to missing risk factors that are geographically dependent.

Clinicians and policymakers might be tempted to use SCORE2 or other risk prediction models, as an alternative to focusing solely on cardiovascular events, to assess spatial distribution of cardiovascular risk. Indeed, the dynamic pattern of events might hinder their effort to develop up-to-date local prevention strategies based on geographical epidemiology. This study help increase the awareness on the risk of biased results when using these tools without any previous validation for a local geographical approach. Further research should focus on replication of ASCVD distribution over the time in specific locations and on the need of employing a valid instrument to spatially predict ASCVD.

### Strengths and limitations

Our study showed some limitations. First, our analysis is based on a closed cohort, thus not allowing to account for immigration over time. A serial cross-sectional design may have improved generalizability. Availability of some covariates only at certain periods and attrition could have reduced the potential to find associations.

It is also noteworthy to consider that our study is susceptible to an ecological fallacy, as results might not fully be applicable to another ecosystem with a different distribution of cardiovascular risk factors. However, this limitation can also be considered as a strength of the study. Indeed, as our results question the use of prediction risk models for a geographical approach and should incentivize policy makers to validate models locally.

This study presents several strengths. We used a population-based cohort design with a rather long follow-up. Limited data are available regarding geographical epidemiology of cardiovascular disease and our study adds to a better understanding of spatial distribution of ASCVD development.

## Conclusion

Using a population-based cohort with a rather long follow-up, we identified clusters of higher and lower incidence of ASCVD, displaying a high variability in incident rate of events, up to ± 2.5% over 10 years compared to the rest of the population.

The geographical distribution of clusters of clinical risk score, mainly driven by alcohol consumption and BMI, was not congruent with that of the ASCVD, limiting their use in identifying areas where ASCVD may develop. Cluster of CVD have been shown to change overtime, underlining the opportunity that prediction tools could offer to assess cardiovascular risk factors dynamically to design up-to-date prevention strategies with a precision public health perspective.

Our results question the use of available prediction risk models for a geographical approach. Further research should focus on replication of ASCVD distribution over the time in specific locations and on the need for clinicians and policymakers of employing a valid instrument to spatially predict ASCVD.

Surprisingly, accounted variables were not associated with clusters of higher incidences of ASCVD. As unaccounted environmental predictors, such as pollution, landscape architecture and noise, might be major drivers of distribution of cardiovascular risk, there is a need to deepen our understanding of these novel risk factors.

### Supplementary Information

Supplementary Information.

## Data Availability

The data that support the findings of this study are available from the *Colaus Study* but restrictions apply to the availability of these data, which were used under license for the current study, and so are not publicly available. Data are however available from the authors upon reasonable request and with permission of the *Colaus study*.
