# Supplementary material for: Spatial analysis of 10-year predicted risk and incident atherosclerotic cardiovascular disease: the CoLaus cohort
Source: Sci Rep. 2024 Feb 27;14:4752. doi: 10.1038/s41598-024-54900-5 (PMC10899582; doi:10.1038/s41598-024-54900-5)
Supplement: Supplementary file 1 — Supplementary Information. [file 41598_2024_54900_MOESM1_ESM.pdf]

Spatial analysis of ten-year predicted risk and incident atherosclerotic cardiovascular disease: the CoLaus cohort

Supplementary material

Guillaume Jordan[1], David Ridder[2,3, 6, 7], Stephane Joost[2,3,6], Peter Vollenweider[1], Martin Preisig[4], Pedro Marques-Vidal[1], Idris Guessous[2,3,6, 7], and Julien Vaucher[1, 5]
1 Department of Medicine, Division of Internal Medicine, Lausanne University Hospital and University of Lausanne, Lausanne, Switzerland
2 Department of Primary Care Medicine, Division of Primary Care Medicine, Geneva University Hospitals, Geneva, Switzerland
3 Laboratory for Biological Geochemistry (LGB), Group of Geospatial Molecular Epidemiology (GEOME), Institute of Environmental Engineering, Ecole Polytechnique Fédérale de Lausanne (EPFL)
4 CEPP, Department of Psychiatry, Lausanne University Hospital and University of Lausanne, Prilly, Switzerland
5 Department of medicine and specialties, Service of internal medicine, Fribourg Hospital and University of Fribourg, Fribourg, Switzerland
6 Geographic Information Research and Analysis in Population Health (GIRAPH)
7 Faculty of Medicine, University of Geneva, Geneva, Switzerland

Corresponding author:
Guillaume Jordan
Department of Medicine,
Lausanne University Hospital (CHUV)
Rue du Bugnon 46
1011 Lausanne - Switzerland
Email: guillaume.jordan@chuv.ch
ORCID N°: 0000-0001-6847-135X

Table of contents

Supplementary method 3
Supplementary results 4
Supplementary tables 6
Supplementary figures 23

Supplementary method

Assessment process in the CoLaus Study

Participants were asked to attend the outpatient clinic at the Centre Hospitalier Universitaire Vaudois (CHUV) in the morning after an overnight fast. They had to take their medication as usual. Data were collected by trained field interviewers in a single visit lasting about 60 minutes. Informed consent was obtained from participants upon their arrival at the study clinic. The first questionnaire mailed with the appointment's letter and completed by the participant prior to the morning visit was then quickly reviewed and a second questionnaire was applied by interview prior to clinical measurements and blood collection. Further details about this cohort study can be found in the original article.[1]

Questionnaire data collection in the CoLaus Study

The first set of questionnaires recorded information on demographic data, socio-economic and marital status, and several lifestyle factors, namely tobacco, alcohol and caffeine consumption, physical activity and mood. Data on smoking included the previous and current smoking status as well as the amount of tobacco smoked (number of cigarettes, cigarillos, cigars or pipes), age of beginning and end (for former smokers). Similarly, data on alcohol consumption included the past and current drinking status as well as the number of alcoholic beverage units (wine, beer and spirits) consumed over the week preceding the interview. Caffeine consumption was assessed by the number of caffeine-containing beverages consumed per day. Personal history of overweight and/or obesity and birth weight were also collected. Finally, the 12-item General Health Questionnaire (GHQ12) was applied in order to screen for the presence of non-psychotic psychiatric disorders.

Low education status was defined considering the lowest educational level achieved when according to a three-level categorization based on questionnaire data. Swiss origin, living in a couple and daily minutes of moderate intensity were also obtained using questionnaires.

Clinical data collection in the CoLaus Study

Body weight and height were measured with participants standing without shoes in light indoor clothes. Body weight was measured in kilograms to the nearest 0.1 kg using a Seca® scale (Hamburg, Germany), which was calibrated regularly. Height was measured to the nearest 5 mm using a Seca® height gauge (Hamburg, Germany). Body mass index (BMI) was defined as weight/height2. Obesity was defined as BMI ≥ 30 kg/m2 and overweight as BMI ≥ 25 kg/m2 and < 30 kg/m2.

BP and heart rate were measured thrice on the left arm, with an appropriately sized cuff, after at least 10 minute rest in the seated position using an Omron® HEM-907 automated oscillometric sphygmomanometer (Matsusaka, Japan) [9]. The average of the last two measurements was used for analyses. Hypertension was defined as a systolic BP (SBP) ≥ 140 mm Hg and/or a diastolic BP (DBP) ≥ 90 mm Hg during the visit and/or presence of anti-hypertensive drug treatment and was considered as known if the subject was aware of this condition.

Prevalent and incident CV events were recorded through a stepwise process.

First, relevant medical records of participants who declared, during the baseline and/or follow-up examinations, to have presented a CVD and/or CVD-related procedure during their lifetime, including MI, angina pectoris, stroke, arrhythmia, cardiomyopathy, coronarography and/or percutaneous transluminal coronary angioplasty (PTCA) and/or coronary stenting, coronary artery bypass grafting (CABG) and peripheral artery disease (PAD), were collected. The records were collected from general practitioners, cardiologists, neurologists and/or hospitals (as appropriate), and encompassed medical and/or surgical notes, laboratory, radiological, echocardiographic and electrocardiographic reports. If necessary, the original coronarography (angiogram) and brain CT/MRI exams were collected. Second, to retrieve events that may not have been mentioned during interviews, the central medical database of the University Hospital of Lausanne, which is the main community hospital in the catchment area of the study, was searched. Participants with hospital records were identified by cross-checking with administrative data and events of interest were detected using the following ICD-10 (International Classification of diseases, Tenth Edition) codes: I20.0, I21.-, I22.-, I24.-, I25.1-, I25.2-, I25.5, I25.6, I25.8, I25.9, I61.-, I62.-, I63.-, I64, I69.1, I69.2, I69.3, I69.4, I69.8, and G45.-. Third, death was established using the population register of the city where the participant was living in case of returned mail, absence of response when calling and/or indication from a relative. Information on cause of death was sequentially collected from: 1) general practitioners; 2) medical database of the hospital where the death occurred (either in Switzerland or abroad); 3) database of the pre-hospital

emergency care unit of the City of Lausanne; 4) database of the University Centres of Forensic Medicine of Lausanne and Geneva; 5) official death certificates from the Swiss federal office of statistics; 6) verbal autopsy with a relative of the dead participant, if all previous steps failed.

Biological data collection in the CoLaus Study

Venous blood samples (50 ml) were drawn after an overnight fast, and most clinical chemistry assays were performed by the CHUV Clinical Laboratory on fresh blood samples whereas Pathway Diagnostics (Los Angeles, CA) measured adiponectin, leptin and insulin [See additional file 1]. Additional aliquots were stored at -80°C.

LDL-cholesterol was calculated with the Friedewald formula only if triglycerides <4.6 mmol/l. Low HDL cholesterol level was defined as <1.0 mmol/L; high HDL cholesterol as ≥ 1.6 mmol/L; high LDL cholesterol was defined as ≥ 4.1 mmol/L and high triglyceride level was defined as ≥ 2.2 mmol/L.

In our analysis, dyslipidemia was defined as low HDL cholesterol and/or high triglyceride and/or LDL cholesterol ≥ 4.1 mmol/L or ≥ 2.6 mmol/L in presence of self-reported history of myocardial infarction, stroke, coronary artery disease or diabetes. Diabetes was defined as fasting plasma glucose ≥ 7.0 mmol/L and/or presence of oral hypoglycemic or insulin treatment. Type 2 diabetes mellitus (T2DM) was defined in case of diabetes without self-reported Type 1 DM. Diabetes was considered as known if the subject was aware of this condition. Impaired fasting glucose (IFG) was defined as fasting plasma glucose between 6.1 and 6.9 mmol/L without anti-diabetic treatment.

Statistical analysis

Relationships between observations are defined by a spatial weight matrix, which assigns identical weights in a critical distance and zero weights outside. Neighborless observations were removed for analyses. In our study, when applying a k-nearest neighbours weighting method, the fixed number of neighbours corresponded to the average neighbours within the distance bands that were used in the primary analysis.

The autocorrelation on the mean squared error between predicted probabilities or risk and observed values (ASCVD) can be thought of as clustering of a Brier score, as the Gi* statistic consist of a ratio of the weighted average of the values in the neighboring locations, to the sum of all values.

In our study, MGWR and GWR models were designed using a bisquare kernel, a golden section bandwidth searching and an Akaike information criterion as an optimization criterion (AICc). Regressions were conducted on individuals without any missing data for any of the covariates used in the most complex model to allow for map comparisons. Additionally, sensitivity analyses were conducted considering participants without any missing data for each of the corresponding models to improve our ability to uncover associations.

In our models we integrated polygenic risk and Mediterranean diet scores. The polygenic risk score is a measure of an individual's genetic susceptibility[2], while the Mediterranean diet score, questionnaire data, reflects adherence to a diet rich in fruits, vegetables, whole grains, legumes, nuts, fish, and olive oil, and low in red and processed meat, saturated fats, and sugar. We used a polygenic score derived by Inouye et al. using 1·7 million single nucleotide polymorphisms where individuals on the top 20th percentile presented a hazard ratio for coronary artery disease of 4·17 (95% CI, 3·97-4·38) compared with those on the bottom 20th percentile. The procedure has been previously reported.[2]

Supplementary results

Reclassification of participants with chronic kidney disease, diabetes or possible familial hypercholesteremia did not change the average risk and its distribution, neither the spatial distribution of predicted risk (*data not shown*).

No global spatial autocorrelation of predicted risk could be detected at the incremental distances. Global Moran’s I value displayed very low indices and high p-values (*supplementary table S5*).

When the age in the SCORE2 algorithm was fixed using the average age of the sample (52 ± 11 at baseline and 58±10 at follow-up) as a constant, high-risk clusters were reinforced and the lower risk ones displayed a different pattern, revealing a west (high risk) to east (low risk) gradient with a small non-hazardous global autocorrelation. (*supplementary figure S3,4)*.

When stratifying by sex, the distribution of many clusters was similar, while some others were unique (s*upplementary figure S5*). The density of clusters was stronger in women, although sex distribution was equivocal in those areas. Adjustment of ASCVD for sex did not change the pattern and distribution of clusters (*supplementary figure S20*).

Supplementary references

1. Firmann, M. *et al.* The CoLaus study: a population-based study to investigate the epidemiology and genetic determinants of cardiovascular risk factors and metabolic syndrome. *BMC Cardiovascular Disorders* **8**, 6 (2008).
2. Inouye, M. *et al.* Genomic Risk Prediction of Coronary Artery Disease in 480,000 Adults: Implications for Primary Prevention. *Journal of the American College of Cardiology* **72**, 1883–1893 (2018).

Supplementary tables

Supplementary table S1. Distribution of independent variables in the participants of the Cohort Colaus-PsyColaus which were included in the analyses

	Baseline (2003-2006)	Follow-up 1 (2009-2012)
**Male sex (n, %)**	2866 (44)	1875 (44)
**Comorbidity[a] (n, %)**	872 (14)	889 (21)
**Lipid modifying drug (n, %)**	678 (11)	693 (16)
**Antihypertensive drug (n, %)**	1107 (18)	1048 (25)
**Body mass index (mean ± SD)**	26 ± 4	26 ± 4
**Weekly units of alcohol consumption (median [IQR])**	4 [0-10]	4 [0-8]
**Living in couple (n, %)**	4127 (66)	2382 (57)
**Low education status (n, %)**	3447 (56)	2219 (53)
**Townsend deprivation index (mean ± SD)**	-.005 ± 2·3	-.08 ± 2·2
**Swiss origin (n, %)**	3683 (60)	2622 (62)
**Anxiety disorder (n, %)**	536 (11)	222 (6)
**Major depressive disorder (n, %)**	980 (21)	724 (20)
**Polygenic risk score (Inouye) (mean ± SD)**	0·5 ± 0·1	0·5 ± 0·1
**Mediterranean diet score (Trichopoulou) (mean ± SD)**		3·9 ± 1·5
**Daily minutes of moderate intensity sport activity (median [IQR])**		0 [0-9]
**Predicted risk with fixed age (median [IQR])**	2·6 [1·8-4·4]	3·8 [2·5-5·2]

Comorbidity[a] defined as presence of either diabetes, chronic kidney failure or possible familial hypercholesterolemia according to the Dutch Lipid Clinic Network diagnostic criteria & Simon Broom criteria (LDL-C > 4·9 mmol/L or Total cholesterol > 7·5 mmol/L)
Description with mean and SD of variables showing a normal distribution and using median and interquartile range for skewed variables
SD, standard deviation; IQR, interquartile range; n, number

Supplementary table S2. Incidence rate differences of incident ASCVD between clusters and the rest of the population

	Incidence rate difference % (IC95%)[a]
**High Geti's-Ord clusters of predicted risk[b]**	
**Baseline - 2003-2006**	1% (-0·01; 2·2)
**Follow-up 1 - 2009-2012**	0·8% (-0·06; 2·4)
**Low Geti's-Ord clusters of predicted risk[b]**	
**Baseline - 2003-2006**	0·2% (-1; 1·5)
**Follow-up 1 - 2009-2012**	-1·2% (-2·7; 0·27)
**High Geti's-Ord clusters of incident ASCVD[b]**	
**Baseline - 2003-2006**	2·5% (1·4; 3·7)
**Follow-up 1 - 2009-2012**	2·1% (0·5; 5·8)
**Low Geti's-Ord clusters of incident ASCVD[b]**	
**Baseline - 2003-2006**	-2·4% (-1·3; -3·5)
**Follow-up 1 - 2009-2012**	-3·3% (-1·8; -5)

Incidence rate differences 95% confidence intervals[a] were computed using Chi-square tests.
Use of a 600 meters bandwidth[b] based on a max-min criterion to avoid isolates (largest of the nearest neighbor distances). Statistical significance of clusters was defined considering a pseudo-p 0·05 based on a permutation inference with 999 permutations.
ASCVD, atherosclerotic cardiovascular events; IC95%, 95% confidence interval

Supplementary table S3. Observed characteristics of participants according to inclusion / exclusion status (2003-2006)

	**Included**	**Excluded**
**N**	6203	530
**Male sex, n (%)**	2861 (46)	328 (62)
**Age [years], mean ± SD**	52 ± 11	54 ± 11
**Current smoker, n (%)**	1670 (27)	142 (27)
**Blood pressure [mmHg], mean ± SD**		
**Systolic**	128 ± 18	132 ± 19
**Diastolic**	79 ± 11	81 ± 11
**Total cholestérol [mmol/L], mean ± SD**	5·6 ± 1	5·7 ± 1
**Hdl-cholestérol [mmol/L], mean ± SD**	1·6 ± 0·4	1·5 ± 0·4
**Ldl-cholestérol [mmol/L], mean ± SD**	3·3 ± 1	3·3 ± 1
**Hypertension, n (%)**	2220 (36)	274 (51)
**Diabetes, n (%)**	351 (6)	62 (12)
**Chronic kidney disease, n (%)**	262 (4)	32 (6)
**Lipid modifying agents, n (%)**	678 (11)	131 (25)
**Antihypertensive agents, n (%)**	1107 (18)	186 (36)

Description with mean and SD of variables showing a normal distribution and using median and interquartile range for skewed variables. SD, standard deviation; IQR, interquartile range; n, number

Supplementary table S4. Performance of SCORE2 & SCORE2-OP combined to predict ten-year incident ASCVD in included participants

	Baseline (2003-2006)	Follow-up 1 (2009-2012)
**N**	6203	4206
**SCORE2 & SCORE2[a], [%], median [IQR]**	2·9 [1·4-5·4]	4 [2-6·9]
**SCORE2 only (Age < 70)**	2·6 [1·3-4·9]	3·3 [1·8-5·4]
**SCORE2-OP only (Age ≥ 70)**	9·8 [7·5-12·8]	11·9 [8·3-15·5]
**Ten-year incident ASCVD (n, %)**	5·7 (357)	6·7 (283)
**Age < 70**	4·9 (280)	5·3 (190)
**Age ≥ 70**	17 (77)	15 (93)
**SCORE2 & SCORE2-OP AUROC (IC95%)**	0·77 (0·75; 0·8)	0·74 (0·71; 0·77)
**SCORE2 only (Age < 70)**	0·76 (0·74; 0·8)	0·73 (0·69; 0·76)
**SCORE2-OP only (Age ≥ 70)**	0·57 (0·51; 0·64)	0·59 (0·53; 0·65)
**No comorbidity**	0·78 (0·75; 0·8)	0·74 (0·71; 0·78)
**Comorbidity[b]**	0·68 (0·63; 0·73)	0·68 (0·62; 0·73)
**Female sex**	0·76 (0·72; 0·8)	0·75 (0·70; 0·80)
**Male sex**	0·75 (0·72; 0·78)	0·76 (0·72; 0·79)
**SCORE2 & SCORE2-OP Brier score**	0·05	0·06
**Inappropriate treatment for current risk[c]**	1762 (28)	1327 (31)

Predicted risks[a] were computed using a combination of SCORE2 for individuals < 70 years and SCORE2-OP for individuals ≥70 years.
Comorbidity[b] defined as presence of either diabetes, chronic kidney failure or possible familial hypercholesterolemia according to the Dutch Lipid Clinic Network diagnostic criteria & Simon Broom criteria (LDL-C > 4·9 mmol/L or Total cholesterol > 7·5 mmol/L)
Inappropriate treatment[c] defined as either absence of lipid modifying or antihypertensive treatment or either uncontrolled blood pressure or inadequate LDL-cholesterol levels for risk category, following the 2021 ESC Guidelines on CVD prevention
ASCVD, atherosclerotic cardiovascular events; IC95%, 95% confidence interval; IQR, interquartile range; n, number

Supplementary table S5. Global spatial autocorrelation of ten-year predicted risk computed using a combination of SCORE2 & SCORE2-OP[a]

	Baseline (2003-2006)	Follow-up 1 (2009-2012)
**Moran's indice – I (pseudo-p)[b]**		
**Bandwidth of 200m**	0·003 (0·088)	0·009 (0·06)
**Bandwidth of 400m**	0·001 (0·185)	0·003 (0·032)
**Bandwidth of 600m**	0·001 (0·198)	0·001 (0·198)
**Bandwidth of 800m**	0·000 (0·32)	0·000 (0·265)
**Bandwidth of 1000m**	0·000 (0·435)	0·000 (0·486)
**Bandwidth of 1200m**	0·000 (0·322)	0·000 (0·126)

Predicted risks[a] were computed using a combination of SCORE2 for individuals < 70 years and SCORE2-OP for individuals ≥70 years.
Statistical significance[b] was defined using on a pseudo-p < 0·05 based on a permutation inference from 999 permutations.
Neighborless observations[c] were removed from analyses.

Supplementary table S6. Performance on a spatial scale of SCORE2 & SCORE2-OP[a]

	Predicted risk, [%], median [IQR]	Incident ASCVD, % (n)
**Whole population**		
**Baseline - 2003-2006**	2·9 [1·4-5·4]	5·7 (357)
**Follow-up 1 - 2009-2012**	4 [2-6·9]	6·7 (283)
**High Geti's-Ord clusters of predicted risk[b]**		
**Baseline - 2003-2006**	3 [1·5-5·7]	6 (265)
**Follow-up 1 - 2009-2012**	4·1 [2·1-7·2]	7 (174)
**Low Geti's-Ord clusters of predicted risk[b]**		
**Baseline - 2003-2006**	2·7 [1·3-5·1]	5·9 (111)
**Follow-up 1 - 2009-2012**	3·9 [2-6·6]	6 (106)
**High Geti's-Ord clusters of incident ASCVD[b]**		
**Baseline - 2003-2006**	2·8 [1·4-5·4]	7 (214)
**Follow-up 1 - 2009-2012**	4·1 [2·1-6·9]	7·7 (166)
**Low Geti's-Ord clusters of incident ASCVD[b]**		
**Baseline - 2003-2006**	2·8 [1·4-5·3]	4·4 (127)
**Follow-up 1 - 2009-2012**	3·9 [1·9-6·8]	5·2 (123)
**High Geti's-Ord clusters of mean squared error between predicted probabilities or risk and observed values[b, c]**		
**Baseline - 2003-2006**	2·9 [1·4-5·4]	6·9 (239)
**Follow-up 1 - 2009-2012**	4·1 [2·1-6·9]	7·7 (165)
**Low Geti's-Ord clusters of mean squared error between predicted probabilities or risk and observed values[b, c]**		
**Baseline - 2003-2006**	2·8 [1·4-5·3]	4·4 (95)
**Follow-up 1 - 2009-2012**	4 [1·9-6·9]	5·1 (99)

Predicted risks[a] were computed using a combination of SCORE2 for individuals < 70 years and SCORE2-OP for individuals ≥70 years.
Use of a 600 meters bandwidth[b] based on a max-min criterion to avoid isolates (largest of the nearest neighbor distances). Statistical significance of clusters was defined considering a pseudo-p 0·05 based on a permutation inference with 999 permutations.
Autocorrelation was also measured on the mean squared error between predicted probabilities or risk and observed values (ASCVD)[c], to identify areas where prediction models lack performance. Because the Gi statistic consist of a ratio of the weighted average of the values in the neighboring locations, to the sum of all values, resulting clusters can be thought of as clusters of Brier score.
ASCVD, atherosclerotic cardiovascular events; IQR, interquartile range; n, number

Supplementary table S7. Model fit metrics for ordinary least squares (OLS) and multiscale geographically weighted regression (MGWR) using predicted risk with age fixed as an outcome

	**1**		**2**		**3**		**4**	
**Type of model**	OLS	MGWR	OLS	MGWR	OLS	MGWR	OLS	MGWR
**Baseline (2003-2006)**								
**R_2**	0·190	0·218	0·191	0·230	0·195	0·235	0·196	0·235
**R_2 adjusted**	0·189	0·207	0·190	0·213	0·193	0·217	0·194	0·217
**AIC**	8920·839	8895·297	8922·165	8892·021	8910·970	8879·718	8909·156	8879·680
**AICc**	8922·851	8896·859	8924·208	8895·484	8913·035	8883·534	8911·234	8883·631
**Follow-up 1 (2009-2012)**								
**R_2**	0·151	0·168	0·153	0·185	0·162	0·199	0·164	0·204
**R_2 adjusted**	0·150	0·160	0·151	0·170	0·158	0·180	0·160	0·183
**AIC**	5619·178	5608·288	5618·898	5603·741	5601·928	5588·740	5601·903	5583·910
**AICc**	5617·159	5608·689	5620·967	5605·303	5604·033	5588·740	5604·077	5586·923

Predicted risks were computed using SCORE2 & SCORE2-OP ESC algorithms. Age was fixed using the mean population age as a constant. Models were designed by incrementally adding independent variables. Independent variables were weekly alcohol consumption and BMI in model 1. Low education status, Townsend deprivation index, Swiss origin and living in a couple were added in model 2 and, additionally, anxiety disorder, major depressive disorder in model 3. Model 4 consisted in the addition of a polygenic risk score at baseline and of a Mediterranean diet score and daily minutes of moderate intensity sport activity at follow-up 1.

Results presented in the table show analyses done on the population with no missing values included in the model 4. Additional analyses using different sizes of population between models were conducted to test for robustness.

MGWR models were conducted using a Gaussian model, an adaptive bisquare kernel, a golden section bandwidth searching and were computed successively on standardized and non-standardized variables. Local collinearity diagnostics were conducted.

AIC, Akaïke information criterion

Supplementary table S8. Bandwidths and parameter estimates for ordinary least squares (OLS) regression and multiscale geographically weighted regression (MGWR) run at baseline (2003-2006) on predicted risk with age fixed

**Model**	1			2			3			4		
**N**	3393			3393			3393			3393		
**Parameters**	BW	β_{GWR} μ±SD	β_{OLS} μ	BW	β_{GWR} μ±SD	β_{OLS} μ	BW	β_{GWR} μ±SD	β_{OLS} μ	BW	β_{GWR} μ±SD	β_{OLS} μ
**Body mass index (BMI)**												
**Raw**		0·14 ±0·00	0·14[a]		0·14 ±0·00	0·14[a]		0·13 ±0·00	0·13[a]		0·13 ±0·00	0·13[a]
**Standardized[b]**	3391	0·30 ±0·09	0·30[a]	3391	0·31 ±0·01	0·31[a]	3391	0·31 ±0·01	0·30[a]	3391	0·30 ±0·01	0·31[a]
**Weekly units of alcohol**												
**Raw**		0·07 ±0·01	0·06[a]		0·06 ±0·02	0·06[a]		0·06 ±0·02	0·06[a]		0·06 ±0·02	0·06[a]
**Standardized[b]**	197	0·24 ±0·07	0·24[a]	172	0·30 ±0·13	0·29[a]	172	0·30 ±0·13	0·28[a]	172	0·30± 0·13	0·29[a]
**Living in couple (Y/N)**												
**Raw**					0·09 ±0·00	0·1		0·07 ±0·00	0·08		0·06±0·01	0·08
**Standardized[b]**				3250	0·02 ±0·01	0·02	3248	0·02 ±0·01	0·02	3290	0·02 ±0·01	0·02
**Low education status (Y/N)**												
**Raw**					0·04 ±0·02	0·05		0·04 ±0·02	0·04		0·03±0·02	0·04
**Standardized[b]**				3391	0·01 ±0·01	0·01	3391	0·01 ±0·01	0·01	3391	0·01 ±0·01	0·01
**Townsend deprivation index**												
**Raw**					0·01 ±0·03	0·02		0·02 ±0·03	0·03[a]		0·01±0·02	0·03
**Standardized[b]**				2002	0·03 ±0·02	0·03	2002	0·03 ±0·03	0·03[a]	2090	0·03± 0·02	0·03
**Swiss origin (Y/N)**												
**Raw**					0·07 ±0·01	0·05		0·06 ±0·01	0·04		0·07±0·01	0·05
**Standardized[b]**				883	0·02 ±0·04	0·01	886	0·02 ±0·03	0·01	886	0·02±0·03	0·01
**Anxiety disorder (Y/N)**												
**Raw**								-0·14 ±0·06	-0·18		-0·13 ±0·06	-0·18
**Standardized[b]**							3335	-0·02 ±0·01	-0·03	3335	-0·02 ±0·01	-0·03
**Major depressive disorder (Y/N)**												
**Raw**								-0·27 ±0·02	-0·24[a]	3368	-0·27 ±0·02	-0·25[a]
**Standardized[b]**							3368	-0·05 ±0·01	-0·05[a]		-0·05 ±0·01	-0·05[a]
**Polygenic risk score (Inouye)**												
**Raw**											0·40± 0·02	0·43
**Standardized[b]**										3391	0·03± 0·00	0·03

Supplementary table S8. Bandwidths and parameter estimates for ordinary least squares (OLS) regression and multiscale geographically weighted regression (MGWR) run at baseline (2003-2006) on predicted risk with age fixed

**Model**	1			2			3			4		
**N**	3393			3393			3393			3393		
**Parameters**	BW	β_{GWR} μ±SD	β_{OLS} μ	BW	β_{GWR} μ±SD	β_{OLS} μ	BW	β_{GWR} μ±SD	β_{OLS} μ	BW	β_{GWR} μ±SD	β_{OLS} μ

Significance for OLS models was defined using a p-value < 0·05[a]. Regarding geographical analyses, β coefficient and significance maps were generated overlapped (data not shown).
Z-transformation on the Y (dependent) and local (independent) variables[b] so that each variable has a mean of 0 and standard deviation of
BW, bandwidth; μ, mean; SD, standard deviation. For further details, check the method section and the tables 5, 6, 7.

Supplementary table S9. Bandwidths and parameter estimates for ordinary least squares (OLS) regression and multiscale geographically weighted regression (MGWR) run at follow-up 1 (2009-2012) on predicted risk with age fixed

Model	1			2			3			4		
N	2098			2098			2098			2098		
**Parameters**	BW	β_{GWR} μ±SD	β_{OLS} μ	BW	β_{GWR} μ±SD	β_{OLS} μ	BW	β_{GWR} μ±SD	β_{OLS} μ	BW	β_{GWR} μ±SD	β_{OLS} μ
**Body mass index (BMI)**												
**Raw**		0·15 ±0·00	0·16[a]		0·14 ±0·00	0·15[a]		0·14 ±0·00	0·15[a]		0·14 ±0·00	0·15[a]
**Standardized[b]**	590	0·31 ±0·06	0·32[a]	590	0·31 ±0·06	0·31[a]	440	0·31 ±0·07	0·31[a]	483	0·3 ±0·07	0·3[a]
**Weekly units of alcohol**												
**Raw**		0·07 ±0·02	0·07[a]		0·06 ±0·02	0·06[a]		0·06 ±0·02	0·06[a]		0·06 ±0·02	0·06[a]
**Standardized[b]**	1792	0·23 ±0·03	0·22[a]	430	0·23 ±0·08	0·22[a]	430	0·22 ±0·08	0·22[a]	430	0·22 ±0·08	0·22[a]
**Living in couple (Y/N)**												
**Raw**					0·16 ±0·01	0·17		0·11 ±0·01	0·11		0·13 ±0·01	0·14
**Standardized[b]**				2092	0·04 ±0·01	0·04	2096	0·03 ±0·00	0·03	2096	0·03 ±0·01	0·03
**Low education status (Y/N)**												
**Raw**					0·06 ±0·01	0·07		0·06 ±0·01	0·07		0·04 ±0·01	0·05
**Standardized[b]**				2096	0·02 ±0·00	0·02	2096	0·01 ±0·00	0·02	2096	0·009 ±0·00	0·01
**Townsend deprivation index**												
**Raw**					0·03 ±0·03	0·03		0·03 ±0·03	0·03		0·03 ±0·03	0·02
**Standardized[b]**				1906	0·03 ±0·03	0·02	1914	0·03 ±0·03	0·03	1869	0·03 ±0·03	0·02
**Swiss origin (Y/N)**												
**Raw**					0·15 ±0·01	0·1		0·16 ±0·02	0·09		0·15 ±0·02	0·08
**Standardized[b]**				700	0·03 ±0·05	0·02	575	0·02 ±0·05	0·02	577	0·02 ±0·05	0·02
**Anxiety disorder (Y/N)**												
**Raw**								-0·54 ±0·03	-0·52[a]		-0·55 ±0·03	-0·54[a]
**Standardized[b]**							2096	-0·05 ±0·00	-0·05[a]	2096	-0·06 ±0·00	-0·06[a]
**Major depressive disorder (Y/N)**												
**Raw**								-0·40 ±0·12	-0·39[a]		-0·40 ±0·10	-0·38[a]
**Standardized[b]**							2096	-0·07 ±0·00	-0·07[a]	1284	-0·07 ±0·02	-0·07[a]
**Mediterranean diet score**												
**Raw**											-0·05 ±0·01	-0·06
**Standardized[b]**										2088	-0·04 ±0·01	-0·04
**Daily minutes of moderate intensity sport activity**												
**Raw**											-0·003 ±0·00	-0·002
**Standardized[b]**										2088	-0·04 ±0·01	-0·03

Supplementary table S9. Bandwidths and parameter estimates for ordinary least squares (OLS) regression and multiscale geographically weighted regression (MGWR) run at follow-up 1 (2009-2012) on predicted risk with age fixed

**Model**	1			2			3			4		
**N**	2098			2098			2098			2098		
**Parameters**	BW	β_{GWR} μ±SD	β_{OLS} μ	BW	β_{GWR} μ±SD	β_{OLS} μ	BW	β_{GWR} μ±SD	β_{OLS} μ	BW	β_{GWR} μ±SD	β_{OLS} μ
**Polygenic risk score (Inouye)**												
**Raw**											-0·15±0·03	-0·17
**Standardized[b]**										2096	0·01±0·01	-0·01

Significance for OLS models was defined using a p-value < 0·05[a]. Regarding geographical analyses, β coefficient and significance maps were generated (data not shown).
Z-transformation on the dependent and independent variables[b], for each variable mean = 0 and standard deviation=1.
BW, bandwidth; μ, mean; SD, standard deviation.

Supplementary table S10. Model fit metrics for binomial logistic regression and binomial geographically weighted regression (GWR) using incident ASCVD as an outcome

**Period**	Baseline (2003-2006)				Follow-up 1 (2009-2012)			
**Model**	1		2		1		2	
**Type of model**	Logistic	GWR	Logistic	GWR	Logistic	GWR	Logistic	GWR
**Fit metrics**								
**Percent deviance explained**	-0·032	-0·037	-0·058	-0·062	-0·048	-0·049	-0·052	-0·053
			-0·062	-0·070	-0·055	-0·063ss	-0·061	-0·067
**Adjusted percent deviance explained**	-0·036	-0·045						
**AIC**	-926·619	464·087	-1378·206	477·549	-758·842	402·382	-830·659	407·110
**AICc**	442·547	464·435	454·935	477·952	380·943	403·090	384·603	407·915

Models run on incident atherosclerotic events were designed using the same variables used in models run on predicted risk with the addition of age, existence of antihypertensive and lipid modifying drug and presence comorbidity defined as presence of either diabetes, chronic renal kidney disease or possible familial hypercholesterolemia. Additionally, in model 2, the risk predicted by SCORE2, and SCORE2-OP algorithm was integrated as an independent variable, fixing age using the mean population age. Additional univariate models were tested.
GWR models were conducted using a binomial model, an adaptive bisquare kernel, a golden section bandwidth searching and were computed successively on standardized and non-standardized variables. Local collinearity diagnostics were conducted.
AIC, Akaïke information criterion; ASCVD: atherosclerotic cardiovascular disease

Supplementary table S11. Model fit metrics OLS regression and MGWR using the mean squared errors between predicted probabilities and observed values as an outcome

**Period**	Baseline (2003-2006)		Follow-up 1 (2009-2012)	
**Model**	1		1	
**Type of model**	OLS	MGWR	OLS	MGWR
**Fit metrics**				
**R_2**	0·046	0·168	0·065	0·105
**R_2 adjusted**	0·042	0·118	0·058	0·083
**AIC**	9498·138	9393·099	5845·229	5823·667
**AICc**	9500·280	9416·871	5847·523	5826·347

Models run on the mean squared errors between predicted probabilities and observed values as an outcome were designed using the same variables used in models run on predicted risk with the addition of age, existence of antihypertensive and lipid modifying drug and presence comorbidity defined as presence of either diabetes, chronic renal kidney disease or possible familial hypercholesterolemia.
MGWR models were conducted using a Gaussian model, an adaptive bisquare kernel, a golden section bandwidth searching and were computed successively on standardized and non-standardized variables. Local collinearity diagnostics were conducted.
AIC, Akaïke information criterion

Supplementary table S12. Bandwidths and parameter estimates for logistic and geographically weighted regression GWR run on ten-year incident ASCVD

**Period**	Baseline (2003-2006)				Follow-up 1 (2009-2012)			
**Model**	1		2		1		2	
**N**	3393		3393		2098		2098	
**Bandwidth (standardized model)**	3387		3379		2096		2092	
**Parameters**	$\beta_{GWR,}$ μ±SD	β_{OLS} μ	$\beta_{GWR,}$ μ±SD	$\beta_{OLS,}$ μ	$\beta_{GWR,}$ μ±SD	β_{OLS} μ	$\beta_{GWR,}$ μ±SD	β_{OLS} μ
**Age**								
**Raw**	0·05±0·01	0·06[a]	0·06±0·01	0·06[a]	0·08± 0·01	0·08[a]	0·08±0·01	0·08[a]
**Standardized[b]**	0·57±0·04	0·59[a]	0·60±0·04	0·63[a]	0·8±0·08	0·8[a]	0·82 0·09	0·84[a]
**Comorbidity (Y/N)**								
**Raw**	0·08±0·09	0·10	-0·7±0·20	-0·65[a]	0·14±0·17	0·25	-0·08±0·11	-0·03
**Standardized[b]**	0·35±0·02	0·03	-0·23±0·04	-0·22[a]	0·08±0·04	0·1	-0·03±0·04	-0·01
**Lipid modifying drug (Y/N)**								
**Raw**								
**Standardized[b]**	0·19±0·16	0·23	0·22±0·16	0·27	0·28±0·09	0·32	0·3±0·04	0·33
	0·06±0·03	0·07	0·07±0·03	0·08	0·11±0·02	0·12	0·11±0·01	0·12
**Antihypertensive drug (Y/N)**								
**Raw**								
**Standardized[b]**	0·36±0·13	0·30	0·34±0·13	0·30	0·19±0·13	0·18	0·16±0·05	0·15
	0·13±0·03	0·11	0·13±0·03	0·11	0·08±0·02	0·08	0·07±0·02	0·06
**Body mass index (BMI)**								
**Raw**								
**Standardized[b]**	0·04±0·01	0·05[a]	0·01±0·01	0·02	0·005±0·00	0·006	-0·005±0·00	-0·01
	0·19±0·03	0·20[a]	0·07±0·03	0·09	0·03±0·00	0·03	-0·02±0·01	-0·03
**Weekly units of alcohol**								
**Raw**								
**Standardized[b]**	0·01±0·01	0·012	-0·01±0·01	-0·01	0·02±0·00	0·02	0·01±0·00	0·01
	0·12±0·04	0·11	-0·06±0·04	-0·06	0·14±0·00	0·12	0·09±0·01	0·08
**Living in couple (Y/N)**								
**Raw**	0·35±0·17	0·35[a]	0·32±0·14	0·31	0·16±0·13	0·13	-0·12±0·08	-0·11
**Standardized[b]**	0·16±0·04	0·16[a]	0·14±0·04	0·16	0·08±0·03	0·07	0·06±0·04	0·05
**Low education status (Y/N)**								
**Raw**								
**Standardized[b]**	0·03±0·10	0·09	0·05±0·10	0·09	0·04±0·1	0·07	0·06±0·05	0·09
	0·03±0·02	0·04	0·04±0·02	0·05	0·02±0·02	0·04	0·03±0·02	0·05
**Townsend deprivation index**								
**Raw**								
**Standardized[b]**	-0·02±0·01	-0·03	-0·02±0·01	-0·03	0·05±0·01	0·03	0·04±0·00	0·03
	-0·05±0·02	-0·06	-0·06±0·01	-0·07	0·09± 0·01	0·06	0·08±0·01	0·06
**Swiss origin (Y/N)**								
**Raw**	0·38±0·09	0·36[a]	0·37±0·07	0·36[a]	0·02±0·12	0·02	0·003±0·05	0·02
**Standardized[b]**	0·17±0·01	0·17[a]	0·17±0·02	0·17[a]	0·005±0·03	0·01	0·001±0·02	0·01
**Anxiety disorder (Y/N)**								
**Raw**	-0·69±0·13	-0·86[a]	-0·67±0·11	-0·82[a]	-0·96±0·53	-1	-0·89±0·3	-0·95
**Standardized[b]**	-0·23±0·02	-0·27[a]	-0·22±0·02	-0·27[a]	-0·22±0·07	-0·23	-0·21±0·07	-0·22
**Major depressive disorder (Y/N)**								
**Raw**	-0·24±0·15	-0·18	-0·20±0·15	-0·12	0·04±0·1	0·28	0·39±0·13	0·33
**Standardized[b]**	-0·09±0·03	-0·07	-0·07±0·03	-0·05	0·13±0·05	0·11	0·15±0·05	0·13
**Polygenic risk score (Inouye)**								
**Raw**								
**Standardized[b]**	2·46±0·38	2·56[a]	2·51±0·30	2·63[a]	2·71±0·82	2·89[a]	2·91±0·40	2·97[a]
	0·35±0·02	0·35[a]	0·35±0·02	0·36[a]	0·38±0·05	0·39[a]	0·39±0·06	0·4[a]
**Mediterranean diet score**								
**Raw**								
**Standardized[b]**					0·04±0·1	0·04	0·05±0·06	0·05
					0·06±0·07	0·06	0·07±0·09	0·07

Supplementary table S12. Bandwidths and parameter estimates for logistic and geographically weighted regression GWR run on ten-year incident ASCVD

**Period**	Baseline (2003-2006)				Follow-up 1 (2009-2012)			
**Model**	1		2		1		2	
**N**	3393		3393		2098		2098	
**Bandwidth (standardized model)**	3387		3379		2096		2092	
**Parameters**	$\beta_{GWR,}$ μ±SD	β_{OLS} μ	$\beta_{GWR,}$ μ±SD	$\beta_{OLS,}$ μ	$\beta_{GWR,}$ μ±SD	β_{OLS} μ	$\beta_{GWR,}$ μ±SD	β_{OLS} μ
**Moderate intensity sport activity**								
**Raw**					-0·01±0·00	-0·007	0·01±0·00	0·01
**Standardized[b]**					-0·23±0·04	-0·2	-0·2±0·03	-0·17
**Predicted risk with fixed age**								
**Raw**								
**Standardized[b]**			0·293±0·29	0·28[a]			0·12±0·02	0·12[a]
			0·57±0·04	0·56[a]			0·26±0·04	0·27[a]

Significance for logistic models was defined using a p-value < 0·05[a]. Regarding geographical analyses, β and significance maps were generated (data not shown); Z-transformation on dependent and independent variables[b].

μ, mean; SD, standard deviation, ASCVD: atherosclerotic cardiovascular events. Comorbidity defined as presence of either diabetes, chronic renal failure or familial hypercholesterolemia. For details, check the method section and tables 5, 6, 7.

Supplementary table S13. Bandwidths and parameter estimates for ordinary least squares (OLS) regression and multiscale geographically weighted regression (MGWR) run on mean squared errors between predicted probabilities and observed values

**Period**	Baseline (2003-2006)			Follow-up 1 (2009-2012)		
**Model**	1			1		
**N**	3393			2098		
**Parameters**	BW	β_{GWR} μ±SD	β_{OLS} μ	BW	β_{GWR} μ±SD	β_{OLS} μ
**Age**						
**Raw**		0·002±0·00	0·002[a]		0·004±0·00	0·004[a]
**Standardized**[b]	1924	0·11±0·04	0·12[a]	636	0·19±0·09	0·18[a]
**Comorbidity (Y/N)**						
**Raw**		0·01±0·01	0·01		0·02±0·06	0·02
**Standardized**[b]	2185	0·01±0·02	0·01	342	0·14±0·11	0·03
**Lipid modifying drug (Y/N)**						
**Raw**						
**Standardized**[b]		0·02±0·16	0·02		0·02±0·00	0·03
	925	0·03±0·07	0·03	2096	0·14±0·00	0·05
**Antihypertensive drug (Y/N)**						
**Raw**						
**Standardized**[b]		0·02±0·00	0·02[a]		0·004±0·03	0·005
	61	0·06±0·23	0·05[a]	2092	0·03±0·01	0·03
**Body mass index (BMI)**						
**Raw**						
**Standardized**[b]		0·002±0·00	0·002[a]		0·000±0·00	0·000
	3047	0·04±0·01	0·04[a]	2096	0·001±0·00	0·003
**Weekly units of alcohol**						
**Raw**						
**Standardized**[b]		0·000±0·00	0·01		0·001±0·00	0·001
	823	0·01±0·05	0·02	2096	0·03±0·00	0·02
**Living in couple (Y/N)**						
**Raw**		0·01±0·00	0·01[a]		0·01±0·00	0·01
**Standardized**[b]	3391	0·03±0·01	0·03[a]	2092	0·02±0·01	-0·03
**Low education status (Y/N)**						
**Raw**						
**Standardized**[b]		0·000±0·00	0·003		0·002±0·00	0·01
	3391	0·01±0·00	0·01	2096	0·01±0·01	0·02
**Townsend deprivation index**						
**Raw**						
**Standardized**[b]	1113	0·000±0·00	-0·001		0·005±0·00	0·003
		0·004±0·04	-0·01	2056	0·04±0·00	0·01
**Swiss origin (Y/N)**						
**Raw**		0·012±0·00	0·01		0·001±0·00	0·003
**Standardized**[b]	3391	0·04±0·00	0·03	2096	0·005±0·01	0·01
**Anxiety disorder (Y/N)**						
**Raw**		-0·02±0·00	-0·02[a]		-0·03±0·00	-0·03
**Standardized**[b]	3391	-0·04±0·00	-0·04[a]	2096	-0·03±0·00	-0·03
**Major depressive disorder (Y/N)**						
**Raw**		-0·01±0·00	- 0·01		0·02±0·02	0·01
**Standardized**[b]	3391	-0·02±0·00	-0·01	1983	0·03±0·02	0·003
**Polygenic risk score (Inouye)**						
**Raw**						
**Standardized**[b]		-0·11±0·00	0·11[a]		0·16±0·00	0·15[a]
	3391	0·08±0·00	0·07[a]	2092	0·1±0·01	0·09[a]
**Mediterranean diet score**						
**Raw**						
**Standardized**[b]					0·003±0·001	0·002
				1798	0·01±0·04	0·01
**Moderate intensity sport activity**						
**Raw**					-0·000±0·00	-0·000
**Standardized**[b]				2096	-0·03±0·00	-0·03

Supplementary table S13. Bandwidths and parameter estimates for ordinary least squares (OLS) regression and multiscale geographically weighted regression (MGWR) run on mean squared errors between predicted probabilities and observed values

**Period**	Baseline (2003-2006)			Follow-up 1 (2009-2012)		
**Model**	1			1		
**N**	3393			2098		
**Parameters**	BW	β_{GWR} $\mu \pm SD$	β_{OLS} μ	BW	β_{GWR} $\mu \pm SD$	β_{OLS} μ

Significance for OLS models was defined using a p-value < 0·05[a]. Regarding geographical analyses, β coefficient and significance maps were generated overlapped (data not shown).
Z-transformation on the Y (dependent) and local (independent) variables[b] so that each variable has a mean of 0 and standard deviation of 1.
BW, bandwidth; μ, mean; SD, standard deviation. For further details, check the method section and the tables 5, 6, 7.

Supplementary figures

Supplementary figure S1. Surface maps of local condition number (A), beta coefficient for weekly alcohol consumption (B) and beta coefficient for body mass index (C) using model 1 and 4 – baseline (2003-2006)

A (Model 1)

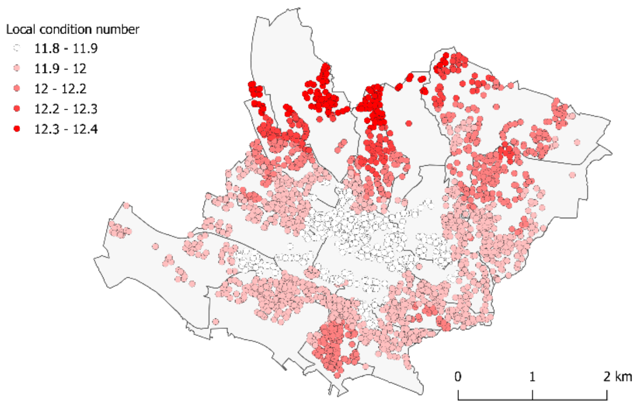

B (Model 1)

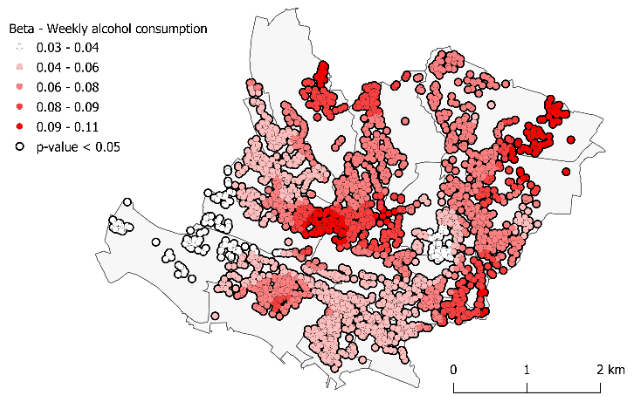

C (Model 1)

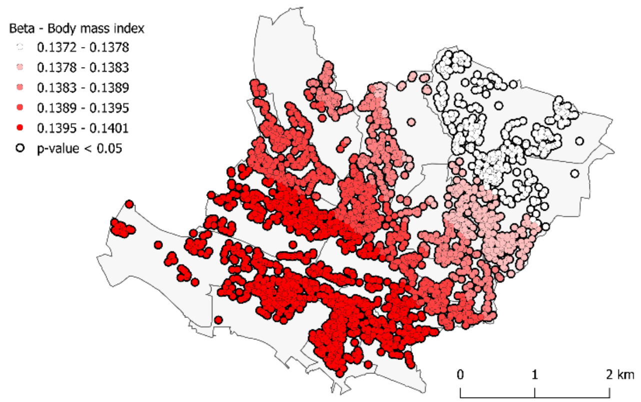

A (Model 4)

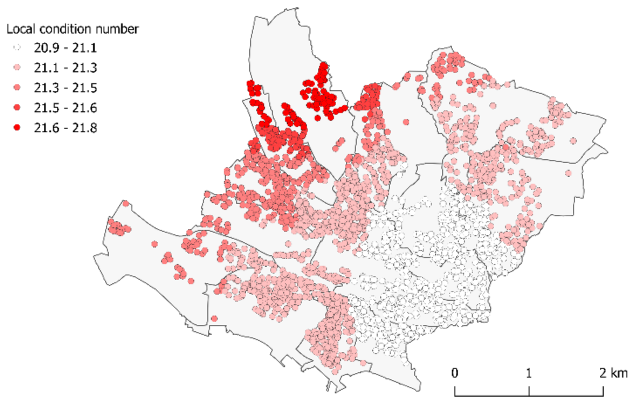

B (Model 4)

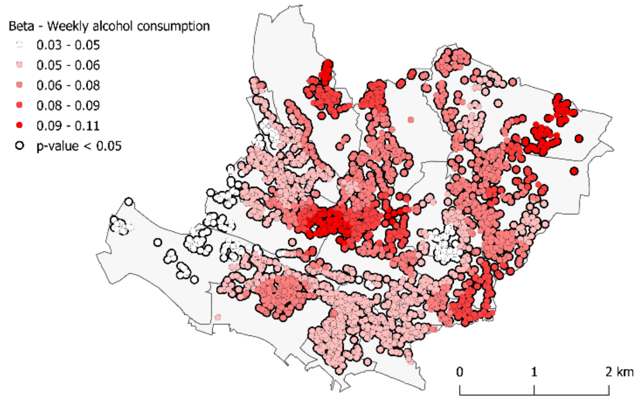

C (Model 4)

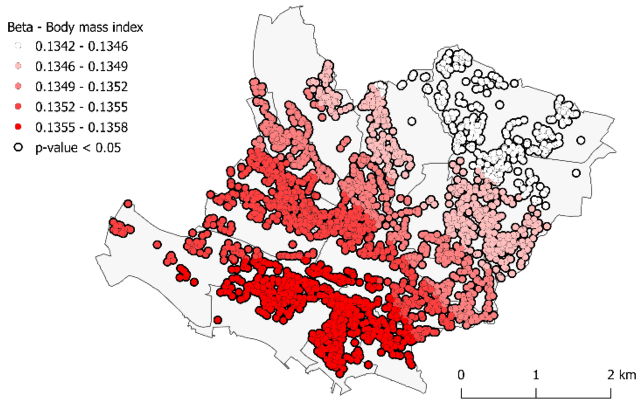

The color markers correspond to a gradient of values with darker tones corresponding to higher values and the lighter ones to lowest values. For each map a singular scale is displayed representing the range of the variable (local condition number, beta coefficient) over the map. Regarding surface maps for beta coefficient, if coefficients display a p-value < 0·05 markers are surrounded by a black line. Model 1 included body mass index and alcohol consumption. Model 4 included additionally living in couple, low education status, Townsend index, Swiss origin, anxiety disorder and major depressive disorder, and a polygenic risk score.

Supplementary figure S2. Surface maps of local condition number (A), beta coefficient for weekly alcohol consumption (B) and beta coefficient for body mass index (C) using model 1 and 4 – follow-up 1 (2009-2012)

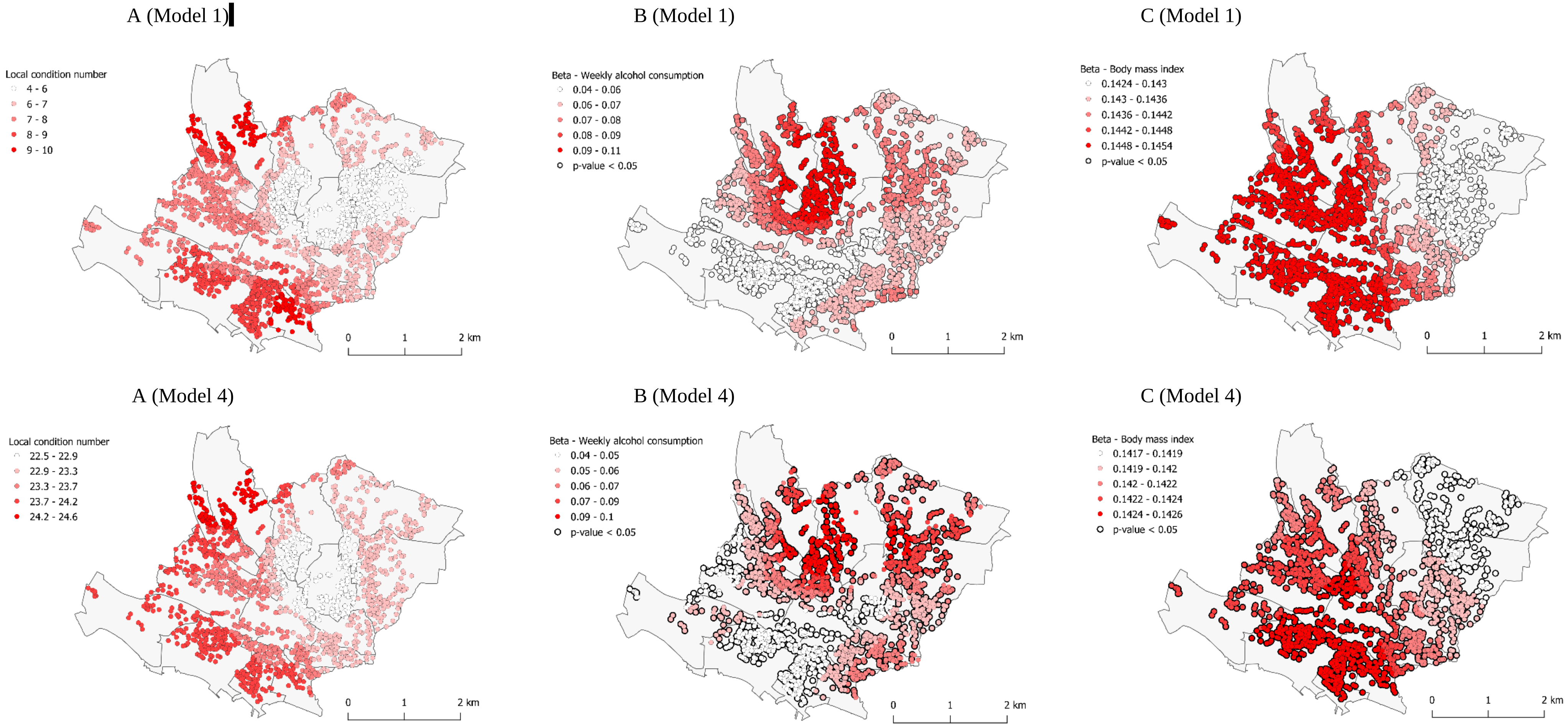

The color markers correspond to a gradient of values with darker tones corresponding to higher values and the lighter ones to lowest values. For each map a singular scale is displayed representing the range of the variable (local condition number, beta coefficient) over the map. Regarding surface maps for beta coefficient, if coefficients display a p-value < 0·05 markers are surrounded by a black line. Model 1 included body mass index and alcohol consumption. Model 4 included additionally living in couple, low education

status, Townsend index, Swiss origin, anxiety disorder and major depressive disorder, a polygenic risk score and at follow-up 1 (2009-2012) a Mediterranean diet score and daily moderate intensity activity.

Supplementary figure S3. Dot maps of predicted risk with each parameter of the risk algorithm fixed iteratively - baseline (2003-2006)

No parameter fixed

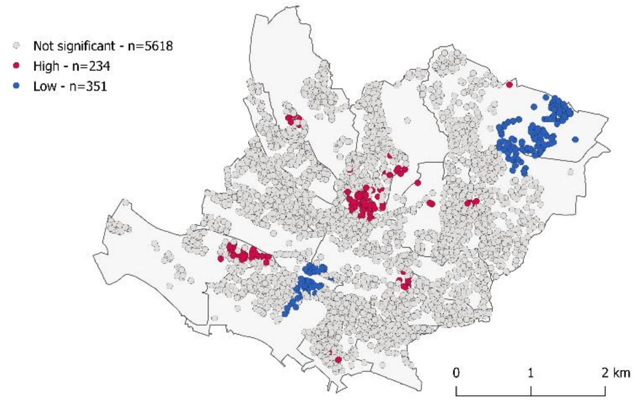

Age fixed

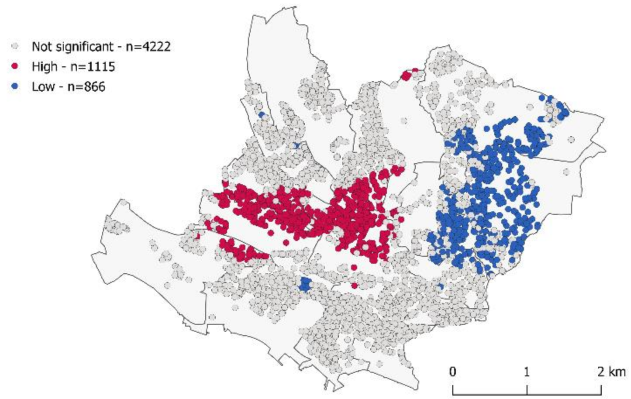

Sex fixed

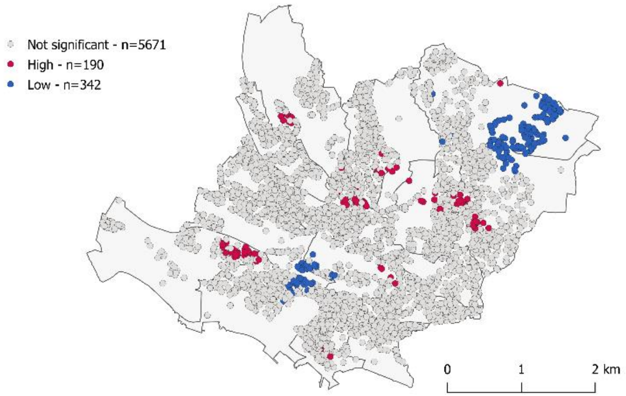

Systolic blood pressure fixed

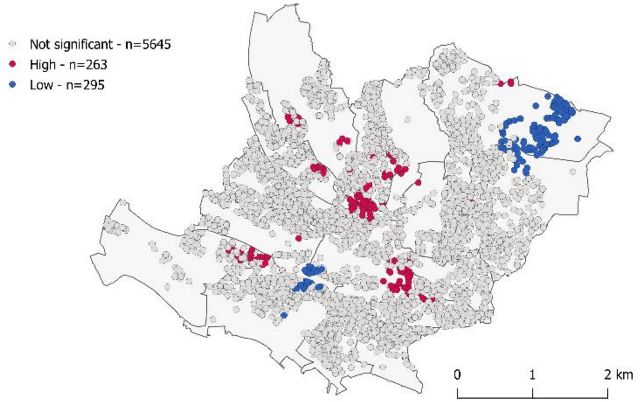

Total cholesterol fixed

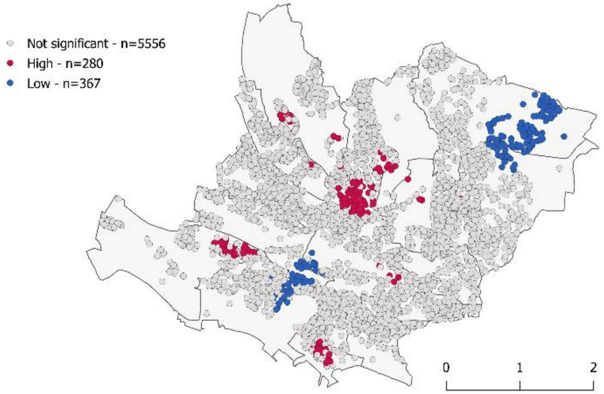

Hdl-cholesterol fixed

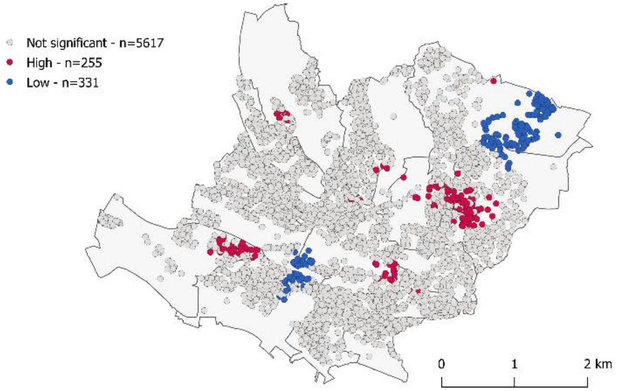

Smoking fixed

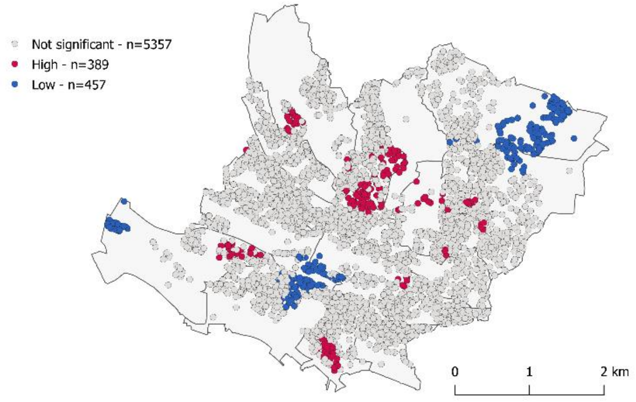

Diabetes fixed

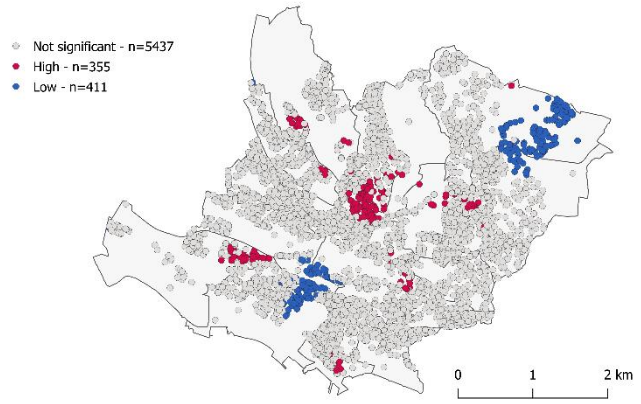

Gi* Getis-Ord for the individuals geo-referenced at their postal address, the CoLaus|PsyCoLaus cohort. White dots show sampling places where the space is neutral (no spatial dependence). Red / Blue dots show individuals with a positive / negative Z score considering a p-value < 0·05 (α=0·05), meaning that high / low values cluster, respectively within a spatial lag of 600 m, are found closer together than expected if the underlying spatial process was random. Pseudo-p 0·05 based on a permutation inference with 999 permutations. Parameters were fixed using the population mean value of the corresponding variable as a constant for continuous variables and a value of zero for binary variables which were coded using either zero or one. Smoking: 0 = non-smoker; 1 = smoker, Sex: 0 = women; 1 = men, Diabetes: 0 = healthy; 1 = Diabetes

Supplementary figure S4. Dot maps of predicted risk with each parameter of the risk algorithm fixed iteratively – follow-up 1 (2009-2012)

No parameter fixed

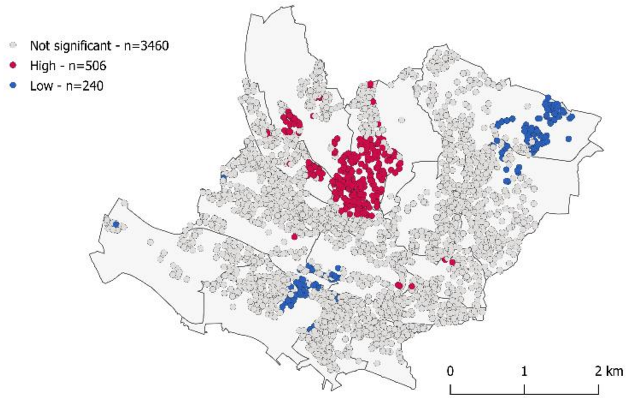

Age fixed

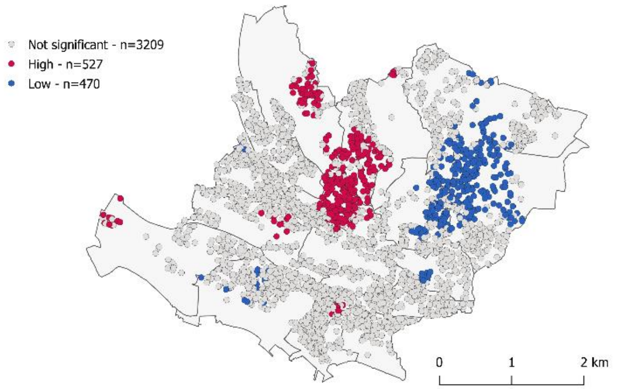

Sex fixed

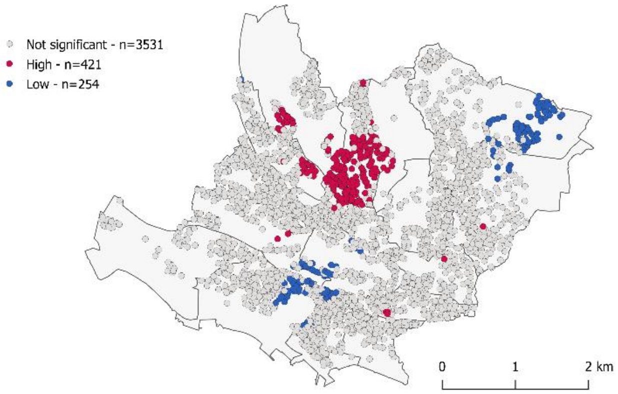

Systolic blood pressure fixed

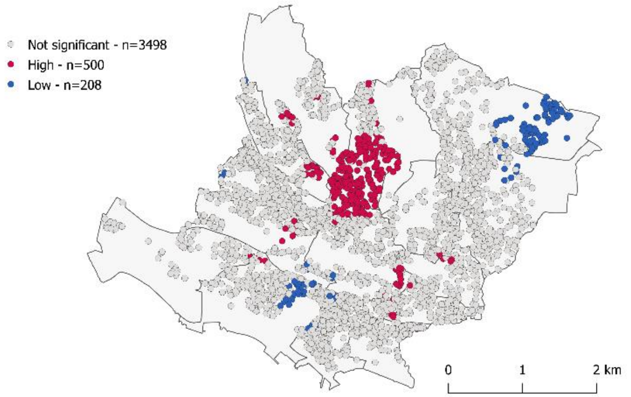

Total cholesterol fixed

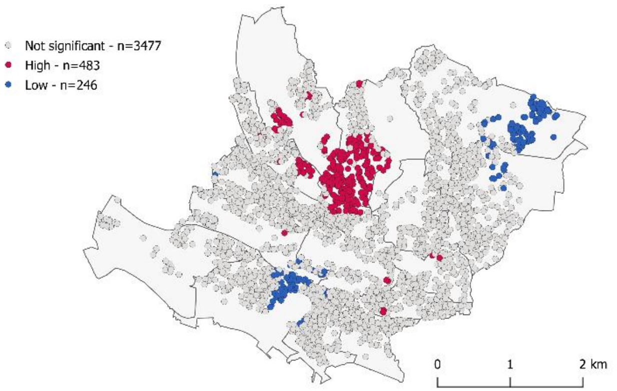

Hdl-cholesterol fixed

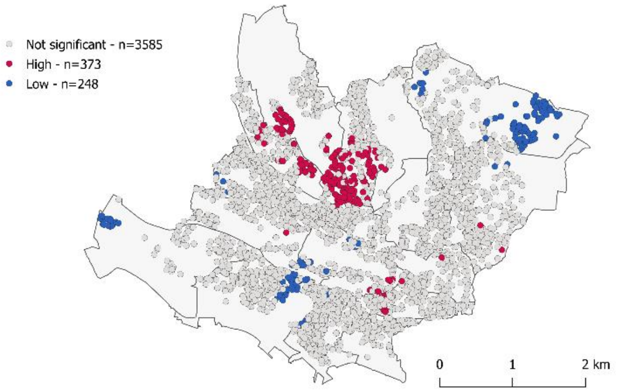

Smoking fixed

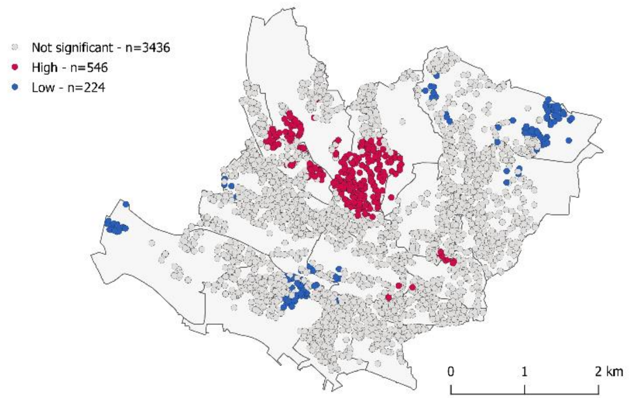

Diabetes fixed

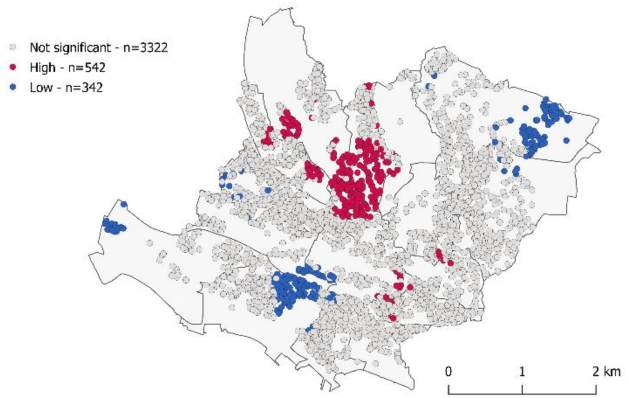

Gi* Getis-Ord for the individuals geo-referenced at their postal address, the CoLaus|PsyCoLaus cohort. White dots show sampling places where the space is neutral (no spatial dependence). Red / Blue dots show individuals with a positive / negative Z score considering a p-value < 0·05 (α=0·05), meaning that high / low values cluster, respectively within a spatial lag of 600 m, and found closer together than expected if the underlying spatial process was random. Pseudo-p 0·05 based on a permutation inference with 999 permutations. Parameters were fixed using the population mean value of the corresponding variable as a constant for continuous variables and a value of zero for binary variables which were coded using either zero or one. Smoking: 0 = non-smoker; 1 = smoker, Sex: 0 = women; 1 = men, Diabetes: 0 = healthy; 1 = Diabetes

Supplementary figure S5. Dot maps of twenty-year incident ASCVD in women (A) and men (B)

A

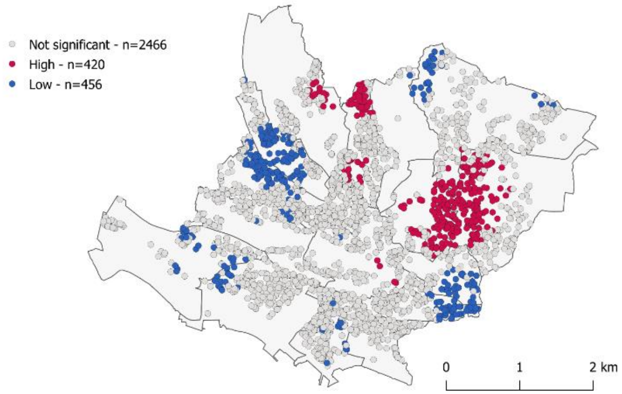

B

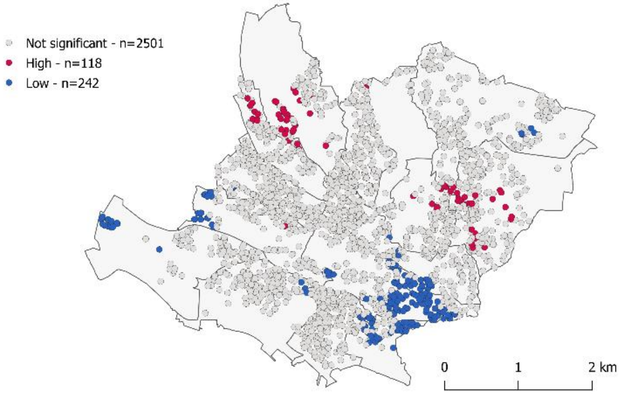

Gi* Getis-Ord for the individuals geo-referenced at their postal address, the CoLaus|PsyCoLaus cohort. White dots show sampling places where the space is neutral (no spatial dependence). Red / Blue dots show individuals with a positive / negative Z score considering a p-value < 0·05 (α=0·05), meaning that high / low values cluster, respectively within a spatial lag of 600 m, are found closer together than expected if the underlying spatial process was random. Pseudo-p 0·05 based on a permutation inference with 999 permutations.

Supplementary figure S6. Flow chart of inclusion

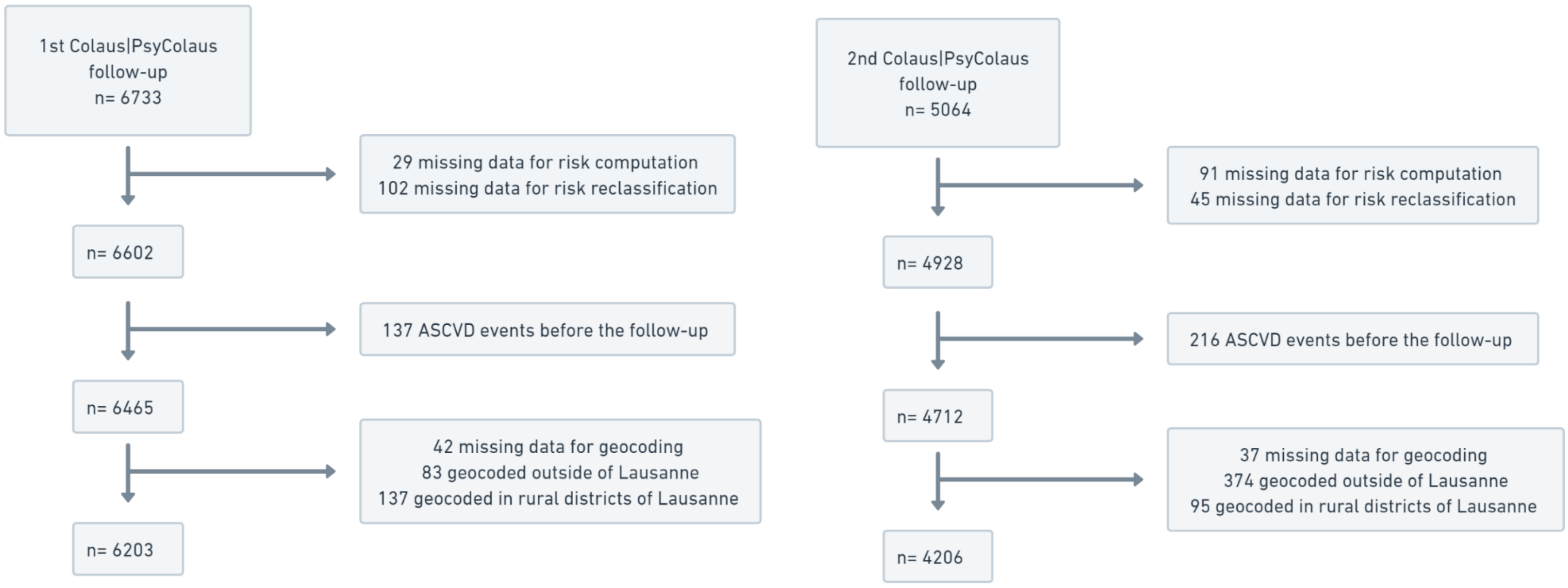

Supplementary figure S7. Percentage of categories of predicted risk as defined by the 2021 ESC Guidelines on CVD prevention stratified by inclusion / exclusion criteria (A) and sex (B)

A

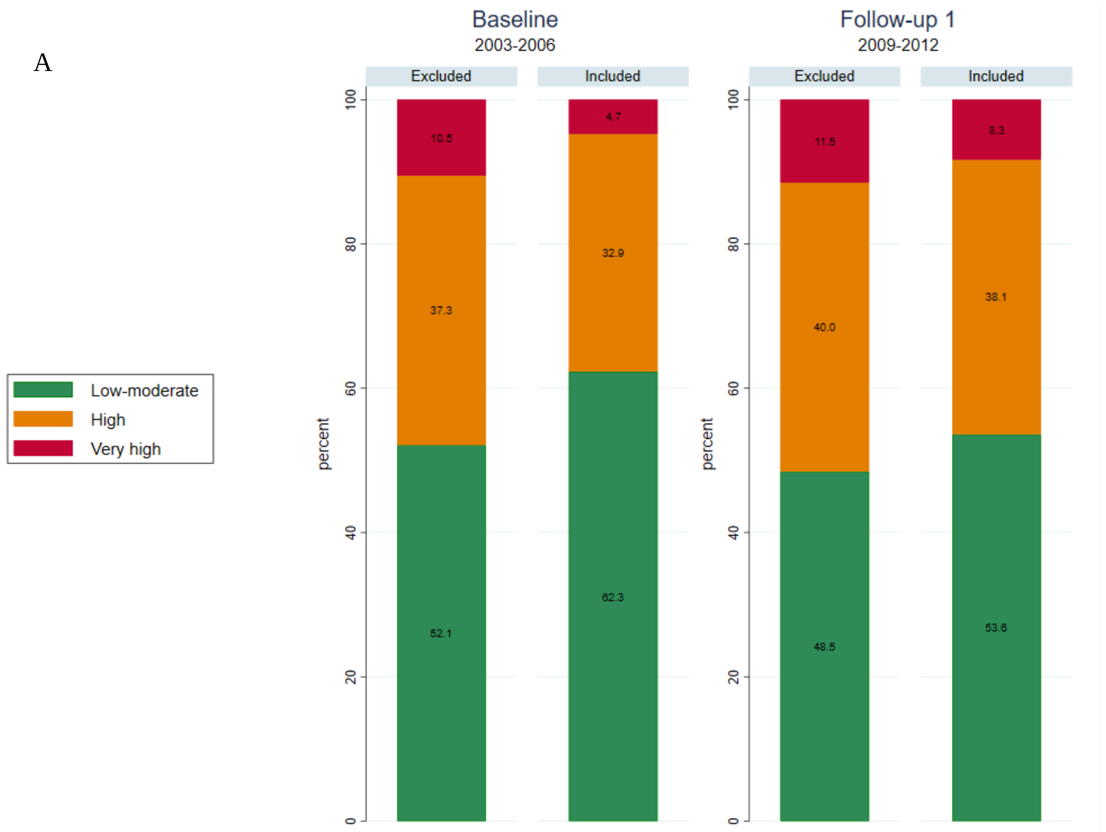

B

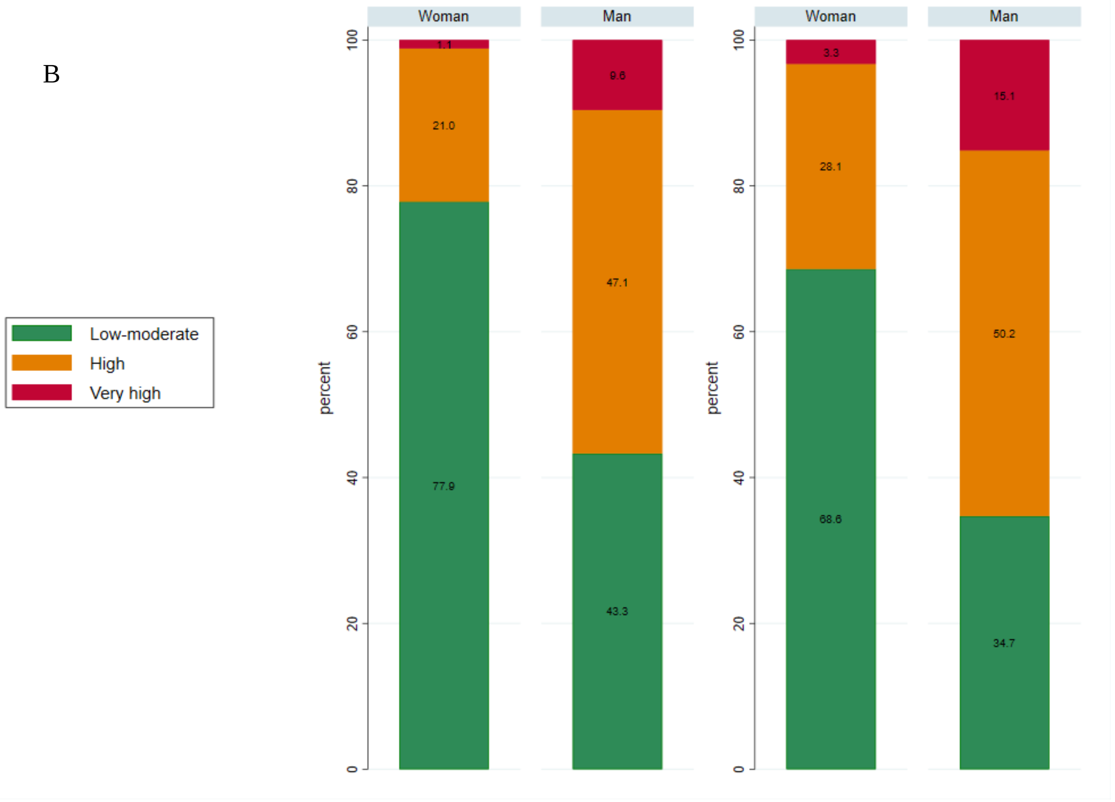

Supplementary figure S8. Dot maps of predicted risk (A), ten-year incident ASCVD (B) and mean squared errors between predicted probabilities and observed values (C) in individuals under 70 years

A (2003-2006)

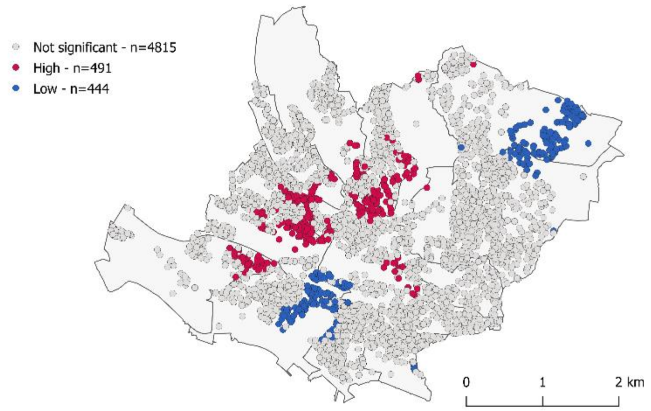

B (2003-2006)

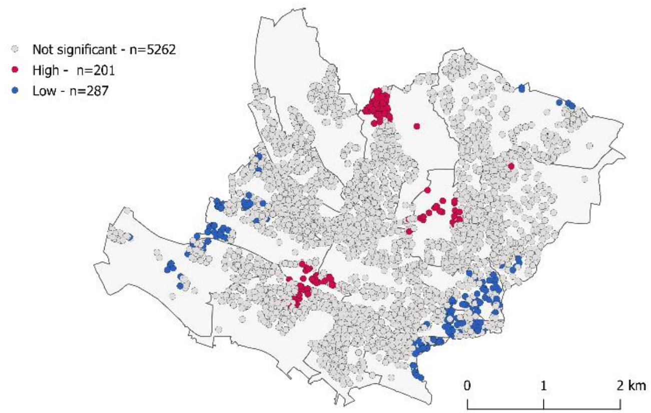

C (2003-2006)

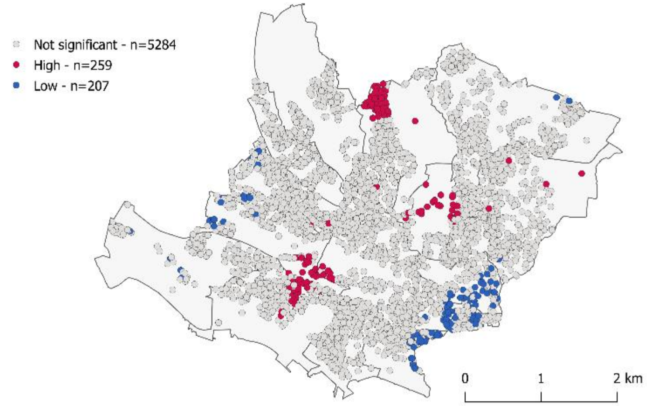

A (2009-2012)

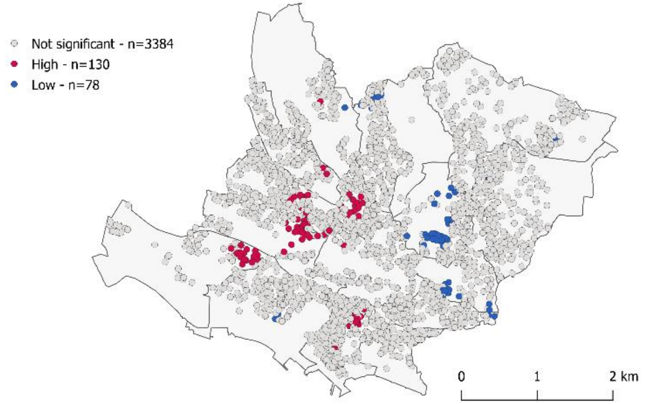

B (2009-2012)

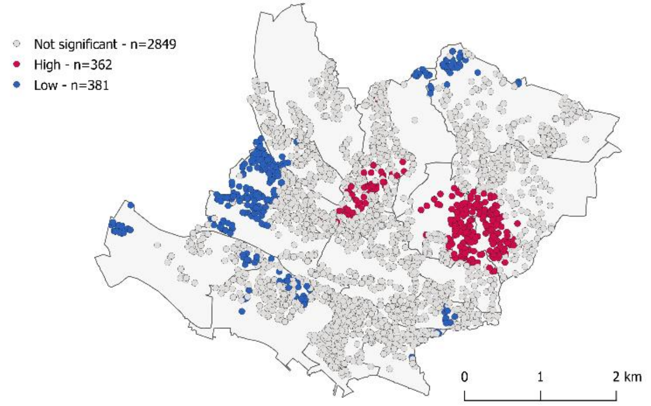

C (2009-2012)

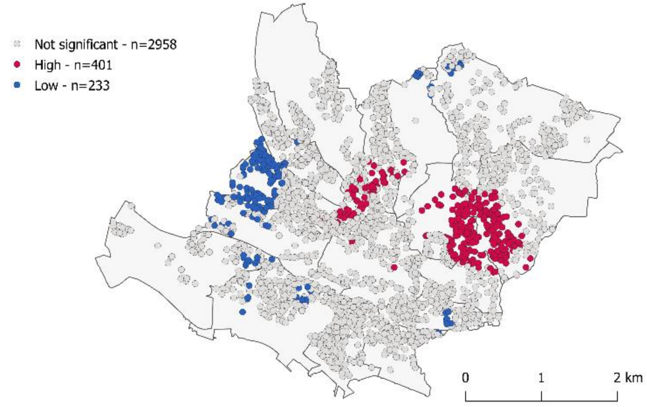

Gi* Getis-Ord for the individuals geo-referenced at their postal address, the CoLaus|PsyCoLaus cohort. White dots show sampling places where the space is neutral (no spatial dependence). Red / Blue dots show individuals with a positive / negative Z score considering a p-value < 0·05 (α=0·05), meaning that high / low values cluster, respectively within a spatial lag of 600 m, are found closer together than expected if the underlying spatial process was random. Pseudo-p 0·05 based on a permutation inference with 999

permutations. The autocorrelation on the mean squared error between predicted probabilities or risk and observed values (ASCVD) can be thought of as clustering of a Brier score, as the Gi* statistic consist of a ratio of the weighted average of the values in the neighboring locations, to the sum of all values.

Supplementary figure S9. Dot maps of predicted risk (A 2003-2006, B 2009-2012), ten-year incident atherosclerotic cardiovascular diseases (ASCVD) after visit (C 2003-2006, D 2009-2012) and mean squared errors between predicted probabilities and ten-year incident ASCVD (E 2003-2006, F 2009-2012) in an apparent healthy population.

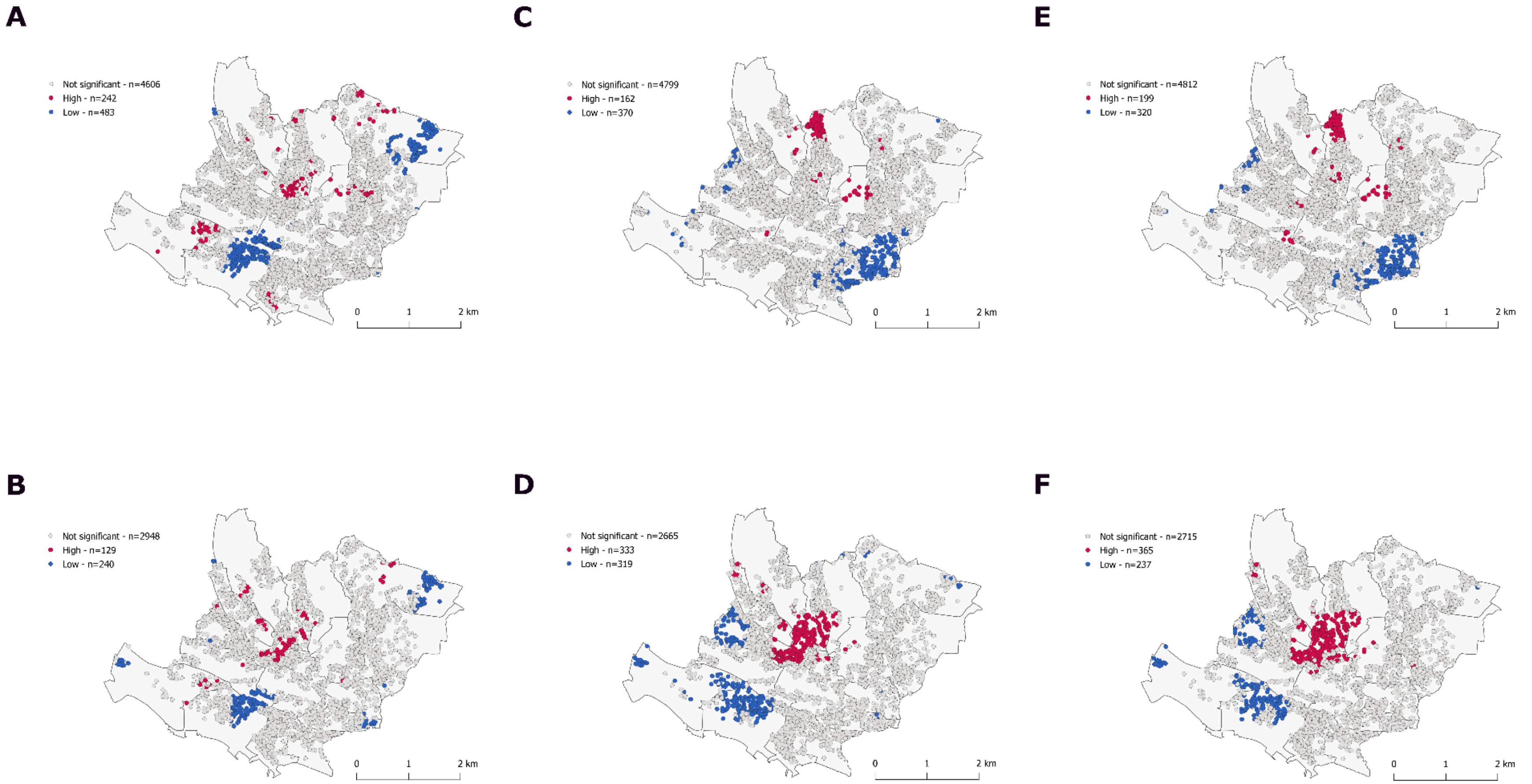

Getis-Ord Gi* statistic for the individuals geo-referenced at their postal address, the CoLaus|PsyCoLaus cohort. White dots correspond to individual with values that do not exhibit spatial dependence. Red / Blue dots indicate individuals with a positive / negative Z score considering a p-value $p<0{\cdot}05$ ($\alpha=0{\cdot}05$). This means that high or low values clusters, respectively, within a spatial lag of 600 m, are found closer together than expected if the underlying spatial process was random. The pseudo-p-value was based on a permutation inference with 999 permutations. The autocorrelation on the mean squared error between predicted probabilities or risk and observed values (ASCVD) can be thought of as clustering of a Brier score, as the Gi* statistic consist of a ratio of the weighted average of the values in

the neighboring locations, to the sum of all values. Individuals with diabetes, chronic kidney disease, possible familial hypercholesterolemia were reclassified. Apparent healthy corresponds to individuals without diabetes, chronic kidney disease, and possible familial hypercholesterolemia.

Supplementary figure S10. Dot maps of predicted risk and ten-year incident ASCVD ten-year after baseline (2003-2006) using baseline geographical coordinates (A), follow-up 1 geographical coordinates (B), follow-up 1 geographical coordinates restricted to individuals who did not move between periods (C)

A (predicted)

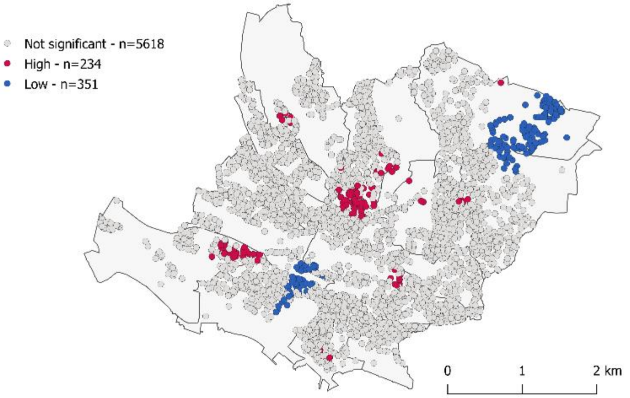

B (predicted)

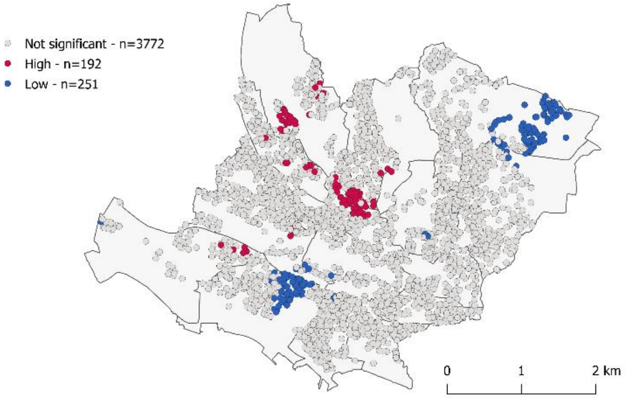

C (predicted)

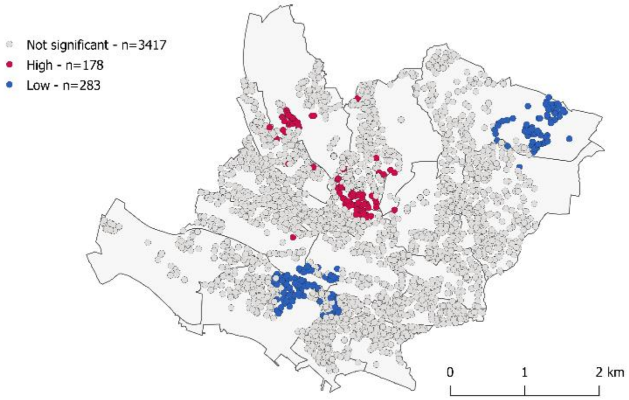

A (ASCVD)

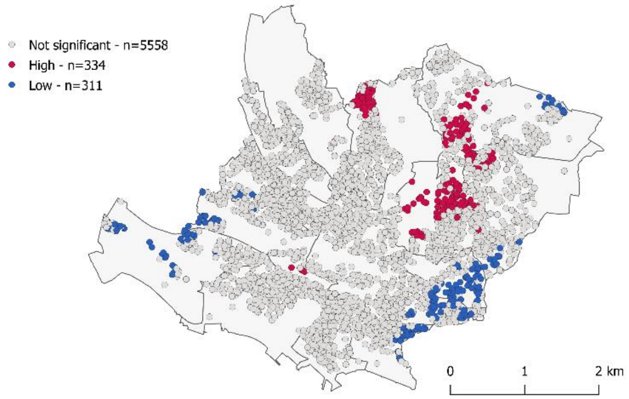

B (ASCVD)

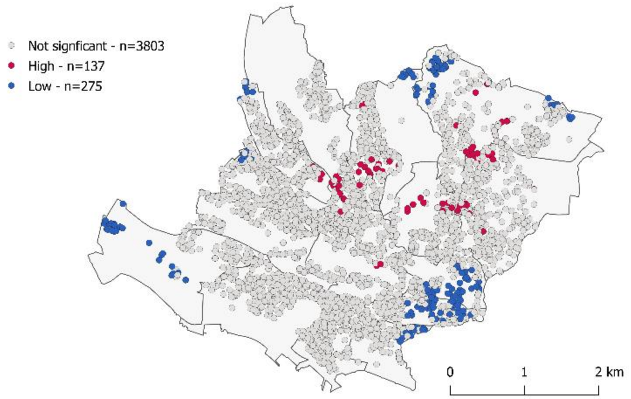

C (ASCVD)

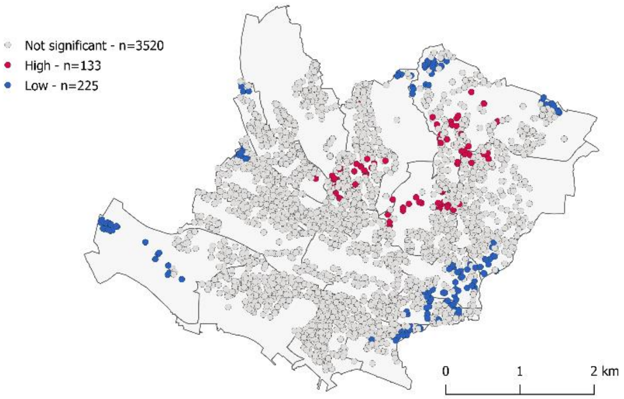

Gi* Getis-Ord for the individuals geo-referenced at their postal address, the CoLaus|PsyCoLaus cohort. White dots show sampling places where the space is neutral (no spatial dependence). Red / Blue dots show individuals with a positive / negative Z score considering a p-value < 0·05 (α=0·05), meaning that high / low values cluster, respectively within a spatial lag of 600 m, and found closer together than expected if the underlying spatial process was random. Pseudo-p 0·05 based on a permutation inference with 999 permutations.

Supplementary figure S11. Dot maps of predicted risk with age fixed unadjusted (A), adjusted for alcohol using a linear regression model (B) and adjusted for alcohol using an MGWR model (C) on individuals without any missing data for alcohol consumption

A (2003-2006)

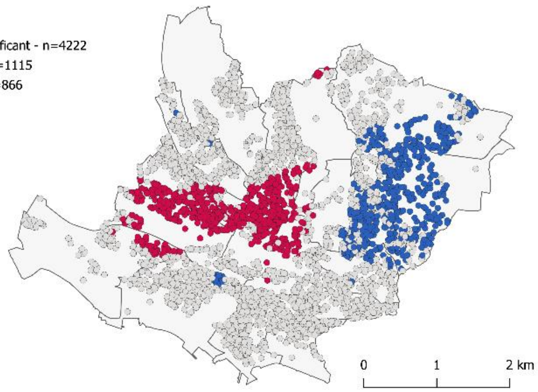

B (2003-2006)

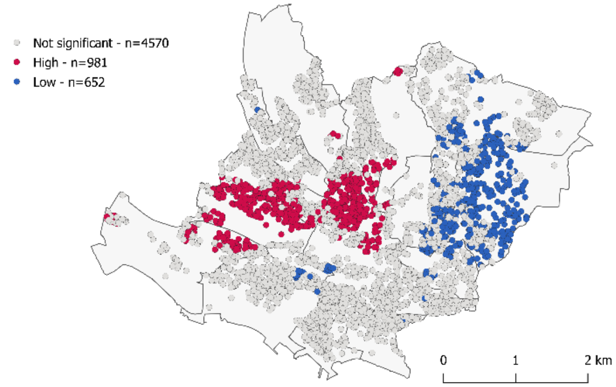

C (2003-2006)

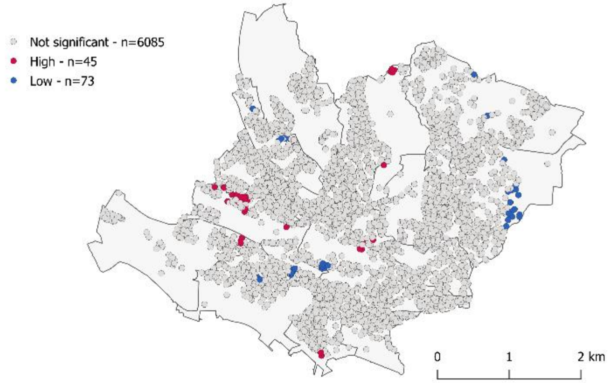

A (2009-2012)

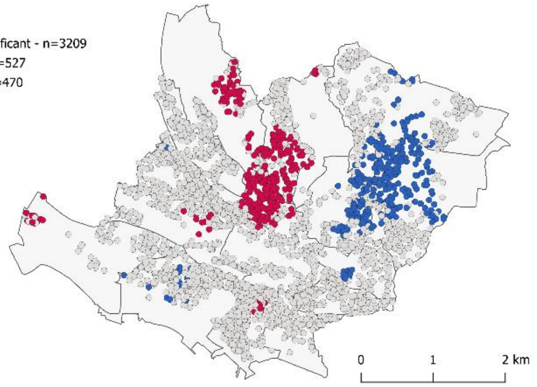

B (2009-2012)

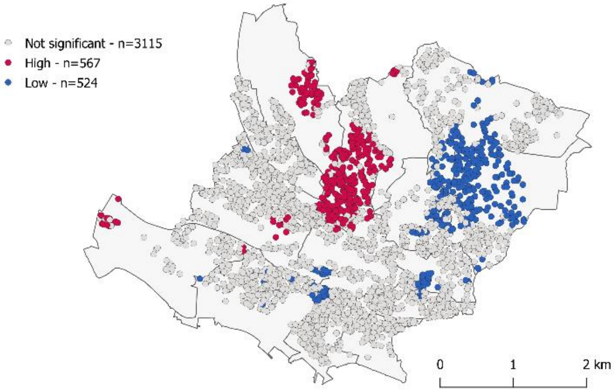

C (2009-2012)

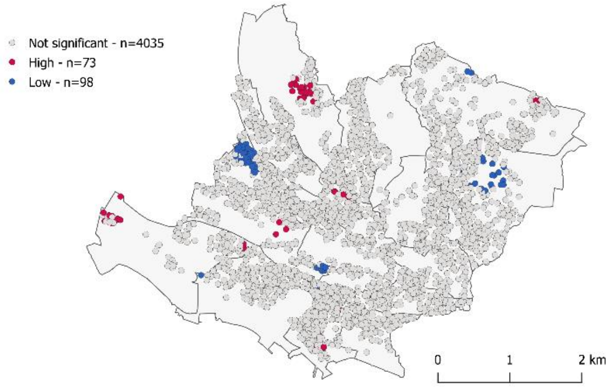

Gi* Getis-Ord for the individuals geo-referenced at their postal address, the CoLaus|PsyCoLaus cohort. White dots show sampling places where the space is neutral (no spatial dependence). Red / Blue dots show individuals with a positive / negative Z score considering a p-value < 0·05 (α=0·05), meaning that high / low values cluster, respectively within a spatial lag of 600 m, are found closer together than expected if the underlying spatial process was random. Pseudo-p 0·05 based on a permutation inference with 999 permutations. Further details regarding models covariates available in the supplementary tables.

Supplementary figure S12. Dot maps of predicted risk with age fixed unadjusted (A), adjusted for body mass index using a linear regression model (B) and adjusted for body mass index using an MGWR model (C) on individuals without any missing data for body mass index

A (2003-2006)

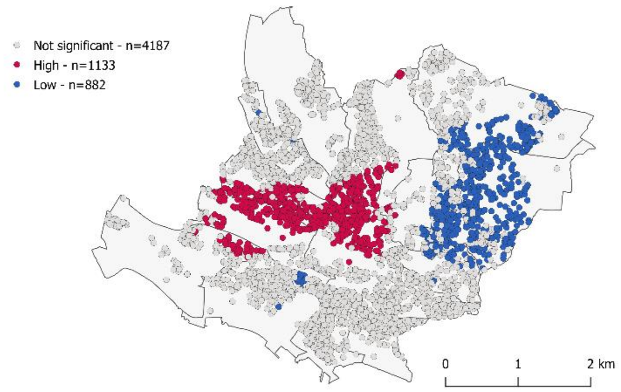

B (2003-2006)

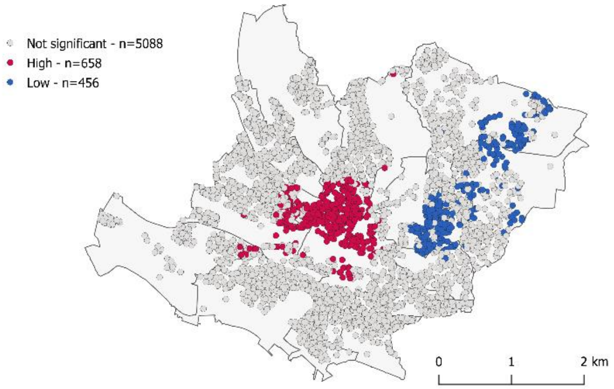

C (2003-2006)

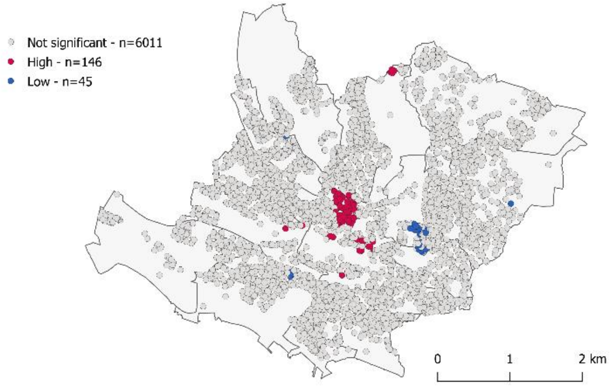

A (2009-2012)

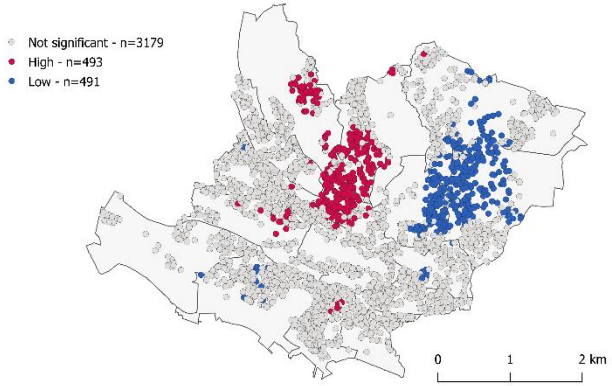

B (2009-2012)

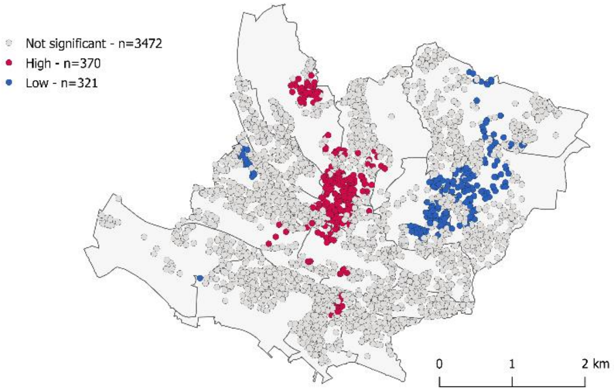

C (2009-2012)

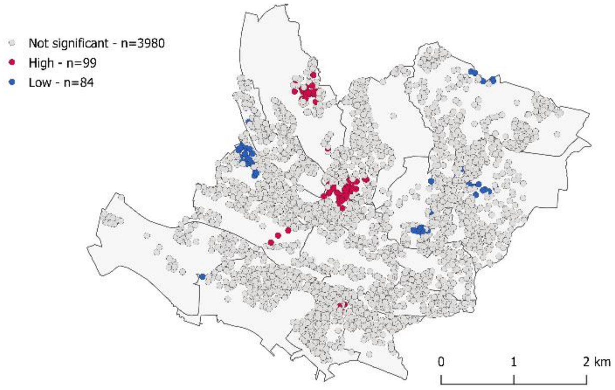

Gi* Getis-Ord for the individuals geo-referenced at their postal address, the CoLaus|PsyCoLaus cohort. White dots show sampling places where the space is neutral (no spatial dependence). Red / Blue dots show individuals with a positive / negative Z score considering a p-value < 0·05 (α=0·05), meaning that high / low values cluster, respectively within a spatial lag of 600 m, are found closer together than expected if the underlying spatial process was random. Pseudo-p 0·05 based on a permutation inference with 999 permutations. Further details regarding models covariates available in the supplementary tables.

Supplementary figure S13. Dot maps of predicted risk with age fixed unadjusted (A), adjusted using model 3 in a MGWR regression (B) and adjusted for model 4 in a MGWR regression (C) on individuals without any missing data for any of the covariates used in model 4

A (2003-2006)

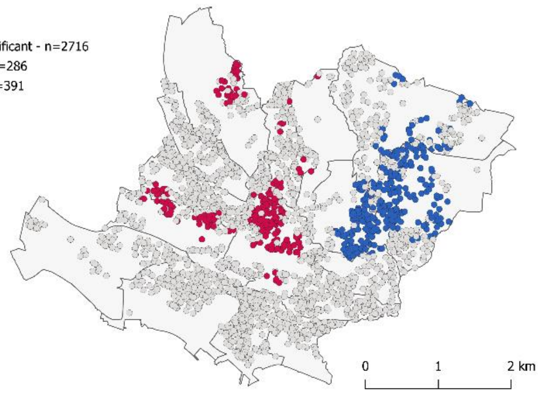

B (2003-2006)

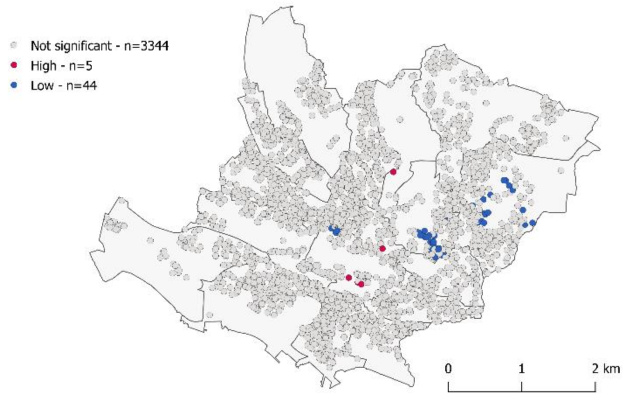

C (2003-2006)

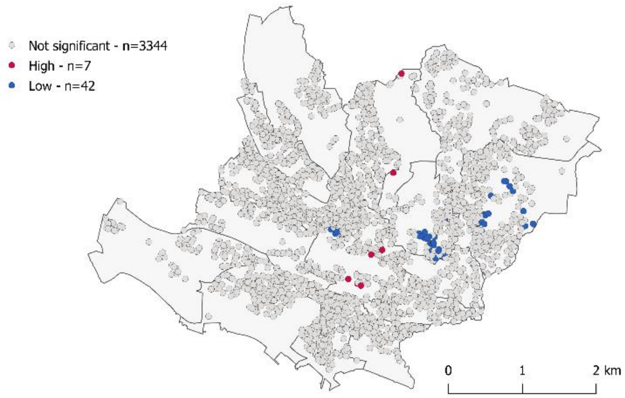

A (2009-2012)

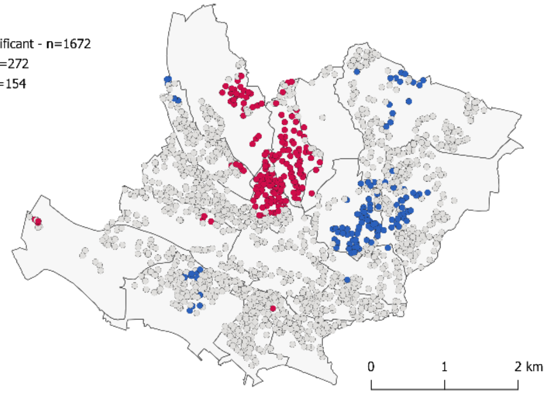

B (2009-2012)

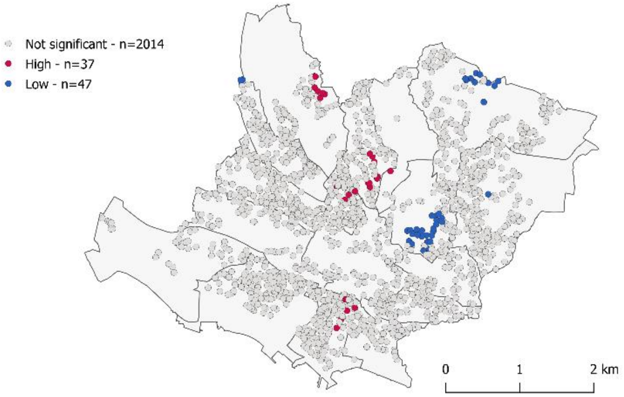

C (2009-2012)

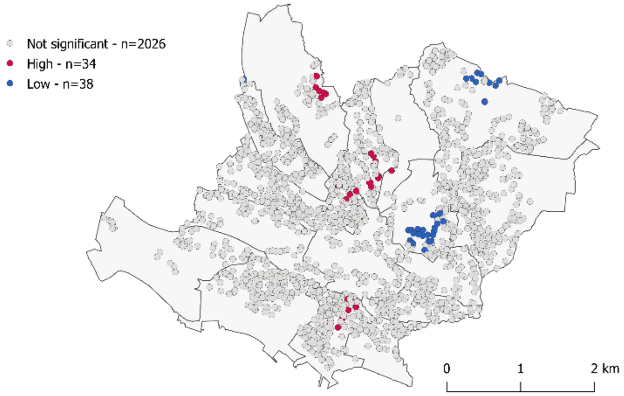

Gi* Getis-Ord for the individuals geo-referenced at their postal address, the CoLaus|PsyCoLaus cohort. White dots show sampling places where the space is neutral (no spatial dependence). Red / Blue dots show individuals with a positive / negative Z score considering a p-value < 0·05 (α=0·05), meaning that high / low values cluster, respectively within a spatial lag of 600 m, are found closer together than expected if the underlying spatial process was random. Pseudo-p 0·05 based on a permutation inference with 999 permutations. Model 3 included body mass index, alcohol consumption, living in couple, low education status, Townsend index,

Swiss origin, anxiety disorder and major depressive disorder. Model 4 included additionally a polygenic risk score and at follow-up 1 (2009-2012) a Mediterranean diet score and daily moderate intensity activity.

Supplementary figure S14. Dot maps of ten-year incident ASCVD unadjusted (A), adjusted using model 1 in a GWR regression (B) and adjusted for model 2 in a GWR regression (C)

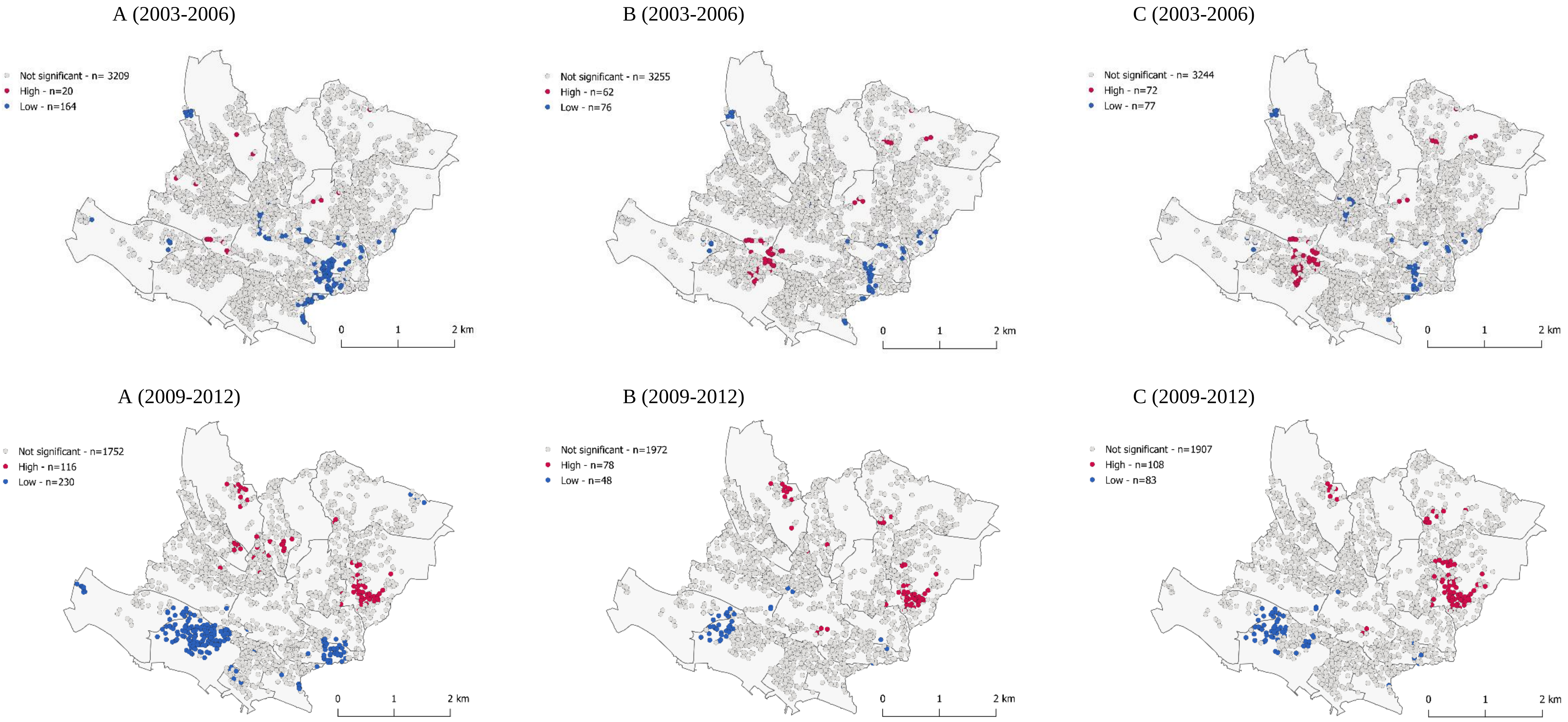

Gi* Getis-Ord for the individuals geo-referenced at their postal address, the CoLaus|PsyCoLaus cohort. White dots show sampling places where the space is neutral (no spatial dependence). Red / Blue dots show individuals with a positive / negative Z score considering a p-value < 0·05 (α=0·05), meaning that high / low values cluster, respectively within a spatial lag of 600 m, are found closer together than expected if the underlying spatial process was random. Pseudo-p 0·05 based on a permutation inference with 999 permutations. Model 1 included age, comorbidity, lipid modifying and antihypertensive drug, body mass index, alcohol

consumption, living in couple, low education status, Townsend index, Swiss origin, anxiety and major depressive disorder, a polygenic risk score and at follow-up 1 a Mediterranean diet score and daily moderate intensity activity. Model 2 included additionally included the predicted risk with age fixed.

Supplementary figure S15. Dot maps of ten-year incident ASCVD unadjusted (A), adjusted using model 1 variables except the polygenic risk score in a GWR regression (B) and adjusted using model 2 variables except the polygenic risk score in a GWR regression (C)

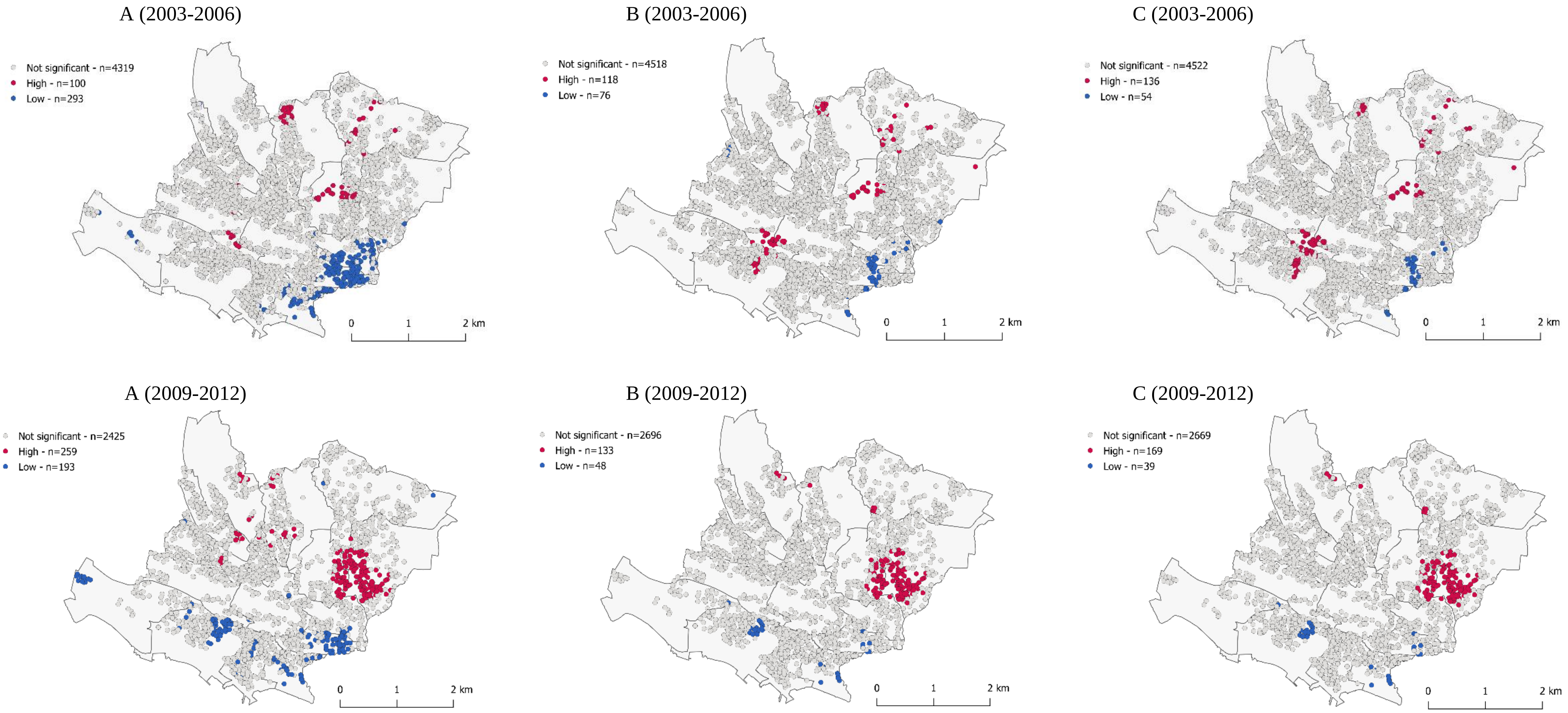

Gi* Getis-Ord for the individuals geo-referenced at their postal address, the CoLaus|PsyCoLaus cohort. White dots show sampling places where the space is neutral (no spatial dependence). Red / Blue dots show individuals with a positive / negative Z score considering a p-value < 0·05 (α=0·05), meaning that high / low values cluster, respectively within a spatial lag of 600 m, are found closer together than expected if the underlying spatial process was random. Pseudo-p 0·05 based on a permutation inference with 999 permutations. Model 1 included age, comorbidity, lipid modifying and antihypertensive drug, body mass index, alcohol consumption, living in couple, low education status, Townsend index, Swiss origin, anxiety and major depressive disorder, a polygenic risk score and at follow-up 1 a Mediterranean diet score and daily moderate intensity activity. Model 2 included additionally included the predicted risk with age fixed.

Supplementary figure S16. Dot maps of ten-year incident ASCVD unadjusted (A), adjusted using age in a GWR model (B)

A (ten-year after 2003-2006)

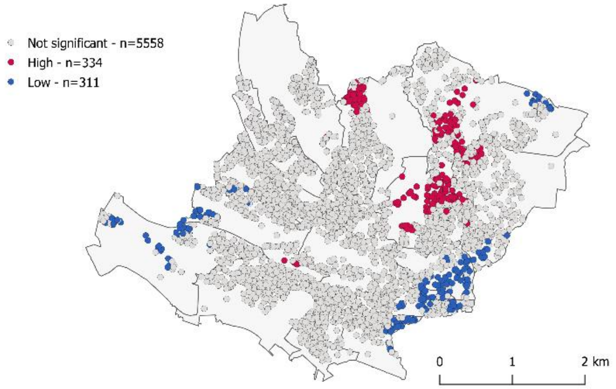

B (ten-year after 2003-2006)

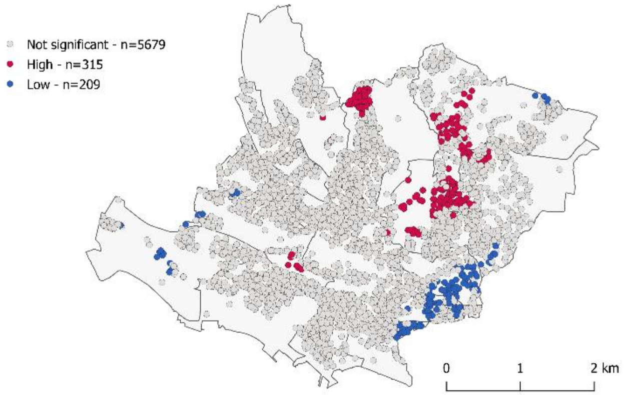

A (ten-year after 2009-2012)

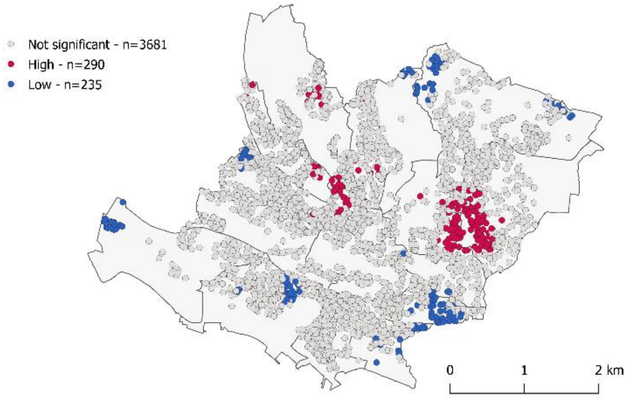

B (ten-year after 2009-2012)

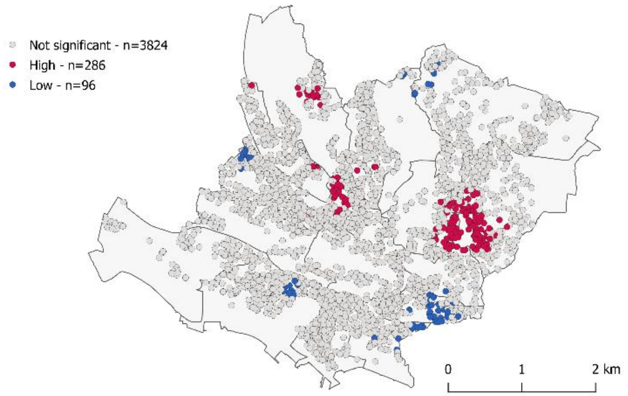

Gi* Getis-Ord for the individuals geo-referenced at their postal address, the CoLaus|PsyCoLaus cohort. White dots show sampling places where the space is neutral (no spatial dependence). Red / Blue dots show individuals with a positive / negative Z score considering a p-value < 0·05 (α=0·05), meaning that high / low values cluster, respectively within a spatial lag of 600 m, are found closer together than expected if the underlying spatial process was random. Pseudo-p 0·05 based on a permutation inference with 999 permutations.

Supplementary figure S17. Dot maps of ten-year incident ASCVD unadjusted (A), adjusted using presence of either diabetes | chronic kidney disease | possible familial hypercholesterolemia as an independent variable in a GWR model (B)

A (ten-year after 2003-2006)

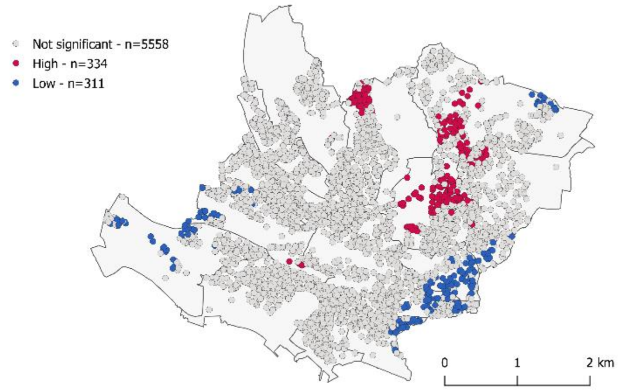

B (ten-year after 2003-2006)

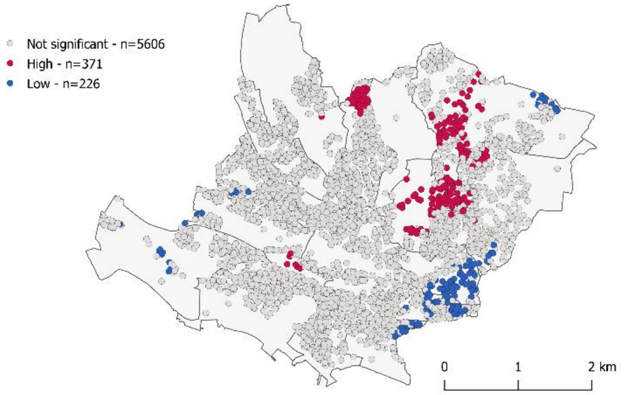

A (ten-year after 2009-2012)

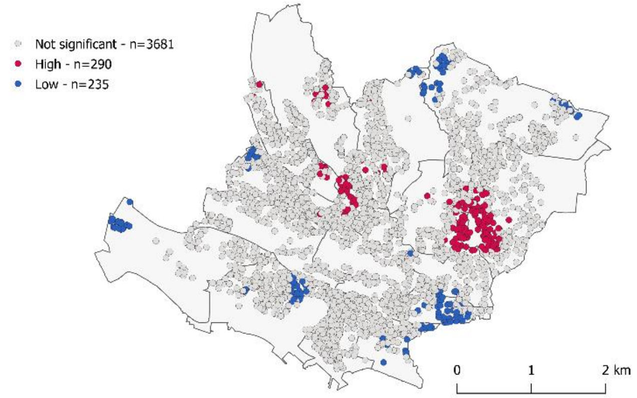

B (ten-year after 2009-2012)

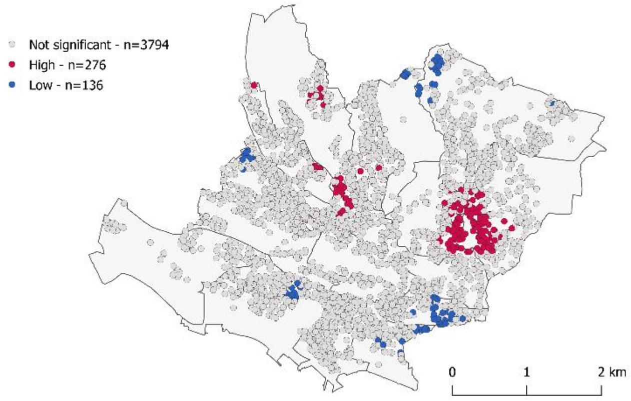

Gi* Getis-Ord for the individuals geo-referenced at their postal address, the CoLaus|PsyCoLaus cohort. White dots show sampling places where the space is neutral (no spatial dependence). Red / Blue dots show individuals with a positive / negative Z score considering a p-value < 0·05 (α=0·05), meaning that high / low values cluster, respectively within a spatial lag of 600 m, are found closer together than expected if the underlying spatial process was random. Pseudo-p 0·05 based on a permutation inference with 999 permutations.

Supplementary figure S18. Dot maps of ten-year incident ASCVD unadjusted (A), adjusted using a polygenic risk score (Inouye) in a GWR model (B)

A (ten-year after 2003-2006)

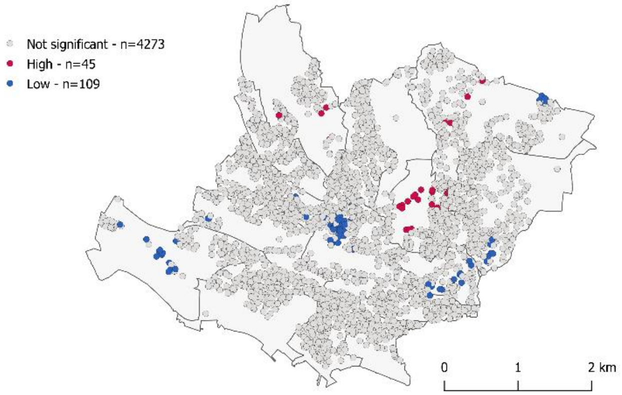

B (ten-year after 2003-2006)

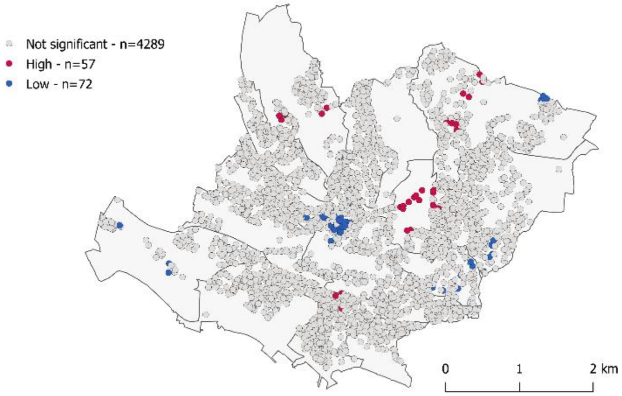

A (ten-year after 2009-2012)

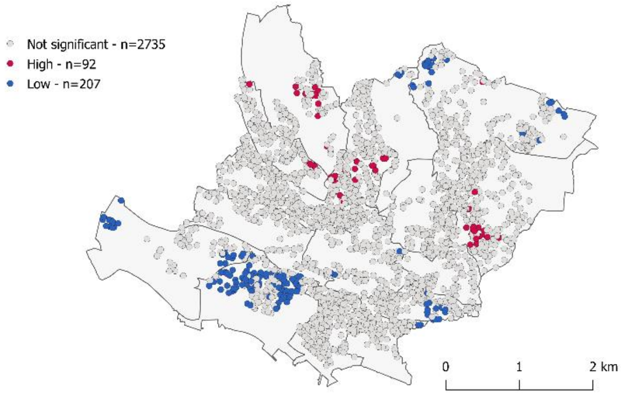

B (ten-year after 2009-2012)

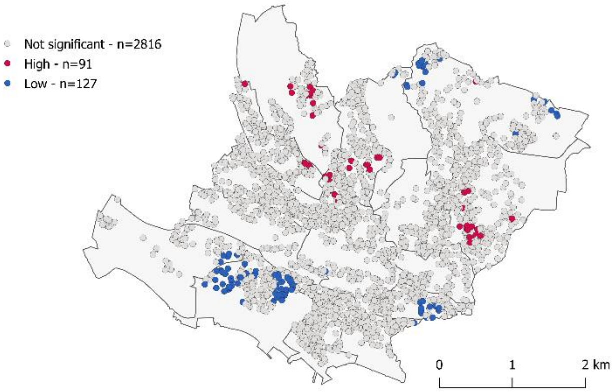

Gi* Getis-Ord for the individuals geo-referenced at their postal address, the CoLaus|PsyCoLaus cohort. White dots show sampling places where the space is neutral (no spatial dependence). Red / Blue dots show individuals with a positive / negative Z score considering a p-value < 0·05 (α=0·05), meaning that high / low values

cluster, respectively within a spatial lag of 600 m, are found closer together than expected if the underlying spatial process was random. Pseudo-p 0·05 based on a permutation inference with 999 permutations.

Supplementary figure S19. Dot maps of ten-year incident ASCVD unadjusted (A), adjusted using predicted risk with hdl-cholesterol fixed in a GWR model (B)

A (ten-year after 2003-2006)

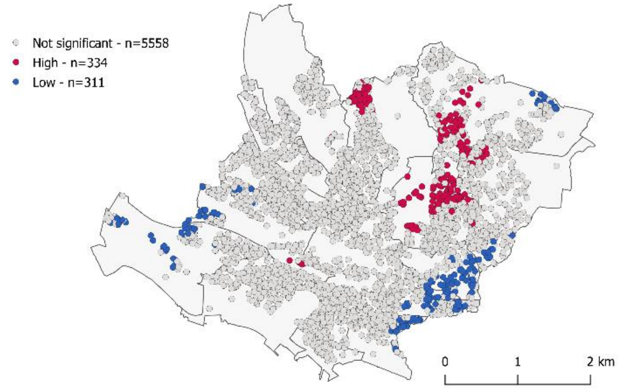

B (ten-year after 2003-2006)

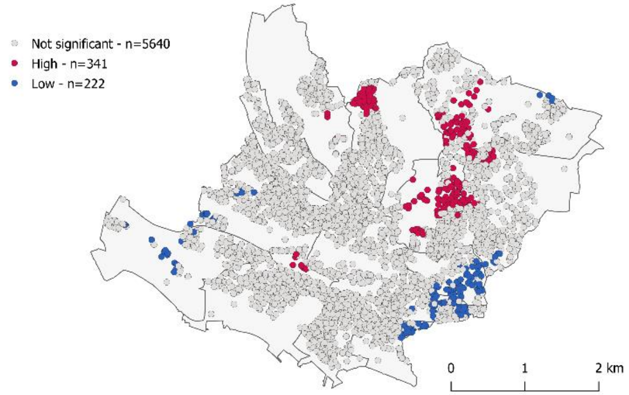

A (ten-year after 2009-2012)

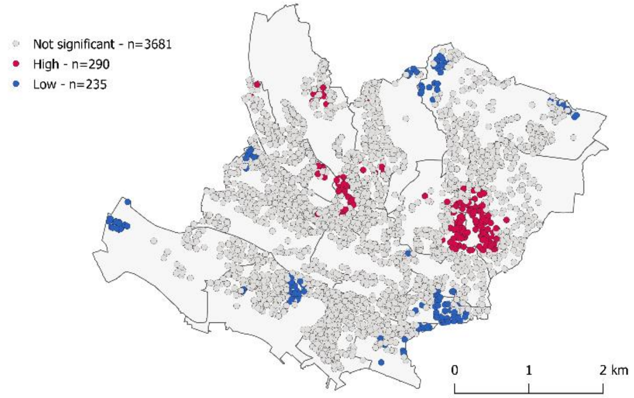

B (ten-year after 2009-2012)

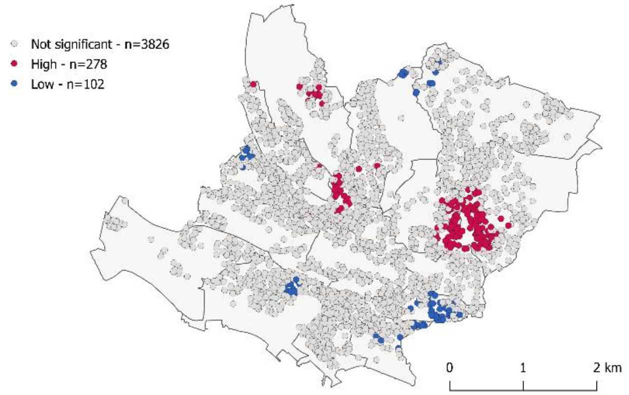

Gi* Getis-Ord for the individuals geo-referenced at their postal address, the CoLaus|PsyCoLaus cohort. White dots show sampling places where the space is neutral (no spatial dependence). Red / Blue dots show individuals with a positive / negative Z score considering a p-value < 0·05 (α=0·05), meaning that high / low values cluster, respectively within a spatial lag of 600 m, are found closer together than expected if the underlying spatial process was random. Pseudo-p 0·05 based on a permutation inference with 999 permutations.

Supplementary figure S20. Dot maps of ten-year incident ASCVD unadjusted (A), adjusted using sex as an independent variable in a GWR model (B)

A (ten-year after 2003-2006)

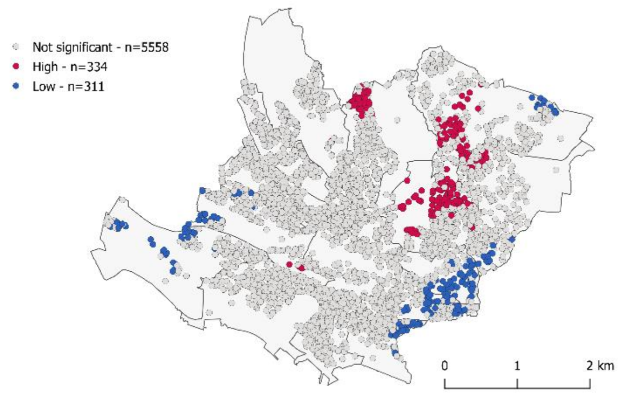

B (ten-year after 2003-2006)

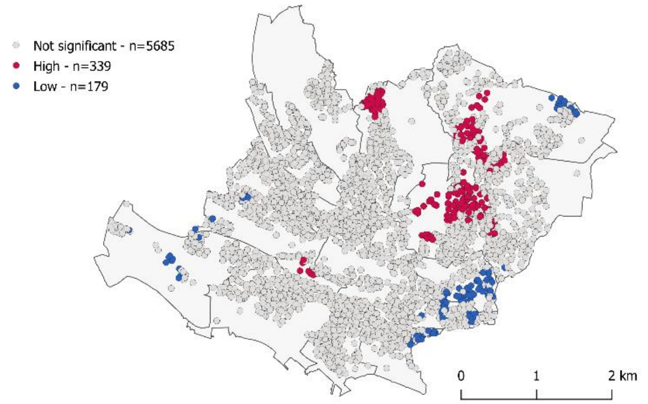

A (ten-year after 2009-2012)

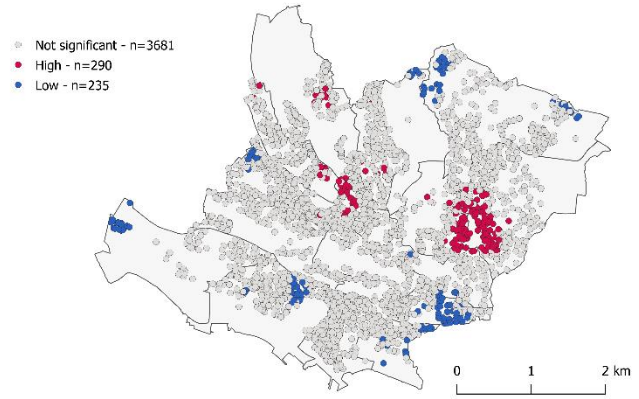

B (ten-year after 2009-2012)

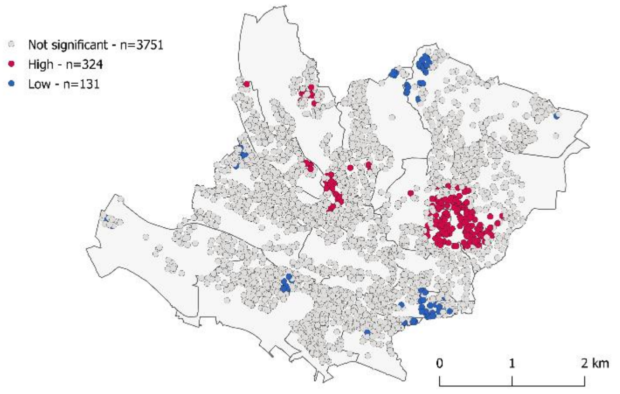

Gi* Getis-Ord for the individuals geo-referenced at their postal address, the CoLaus|PsyCoLaus cohort. White dots show sampling places where the space is neutral (no spatial dependence). Red / Blue dots show individuals with a positive / negative Z score considering a p-value p<0·05 (α=0·05), meaning that high / low values cluster, respectively within a spatial lag of 600 m, are found closer together than expected if the underlying spatial process was random. Pseudo-p 0·05 based on a permutation inference with 999 permutations.

Supplementary figure S21. Dot maps of mean squared errors between predicted probabilities and observed values unadjusted (A), adjusted using model 1 in a MGWR regression (B)

A (2003-2006)

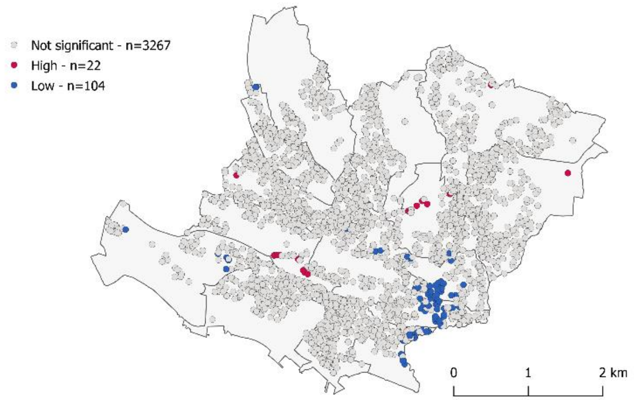

B (2003-2006)

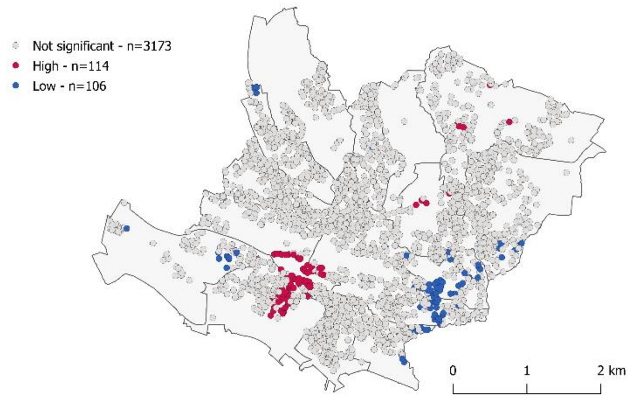

A (2009-2012)

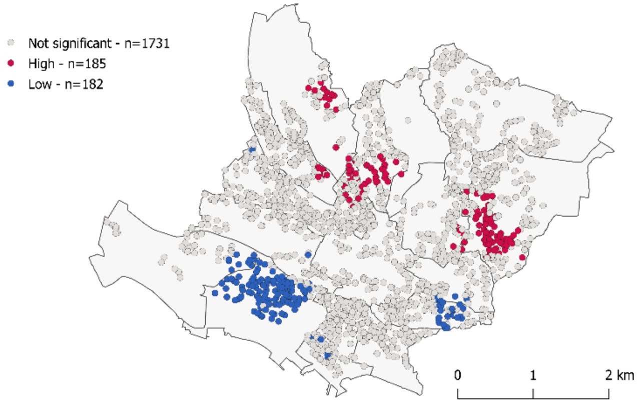

B (2009-2012)

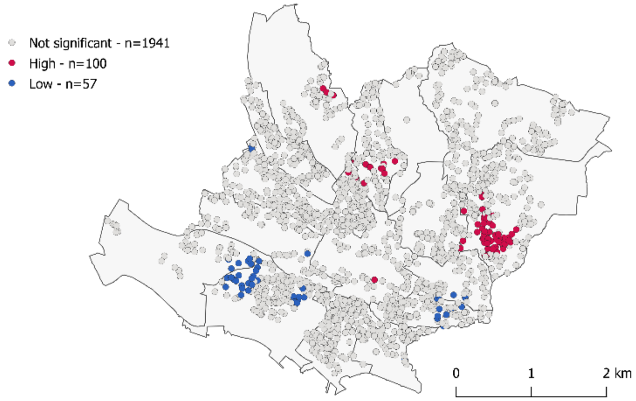

Gi* Getis-Ord for the individuals geo-referenced at their postal address, the CoLaus|PsyCoLaus cohort. White dots show sampling places where the space is neutral (no spatial dependence). Red / Blue dots show individuals with a positive / negative Z score considering a p-value < 0·05 (α=0·05), meaning that high / low values cluster, respectively within a spatial lag of 600 m, are found closer together than expected if the underlying spatial process was random. Pseudo-p 0·05 based on a permutation inference with 999 permutations. The autocorrelation on the mean squared error between predicted probabilities or risk and observed values (ASCVD) can be thought of as clustering of a Brier score, as the Gi* statistic consist of a ratio of the weighted average of the values in the neighboring locations, to the sum

of all values. Model 1 included age, comorbidity, lipid modifying and antihypertensive drug, body mass index, alcohol consumption, living in couple, low education status, Townsend index, Swiss origin, anxiety and major depressive disorder, a polygenic risk score and at follow-up 1 a Mediterranean diet score and daily moderate intensity activity.

Supplementary figure S22. Dot maps of mean squared errors between predicted probabilities and observed values unadjusted (A), adjusted using model 1 variables except the polygenic risk score in a MGWR regression (B)

A (2003-2006)

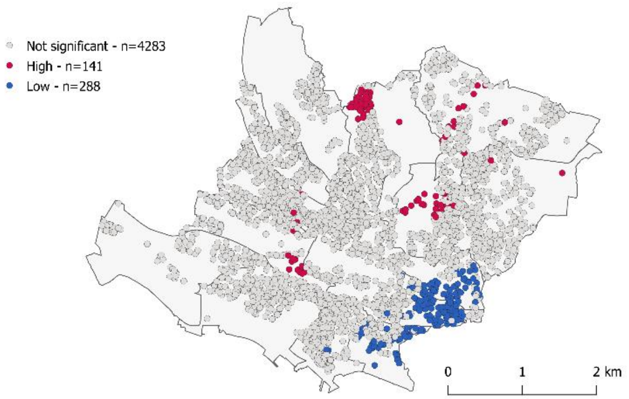

B (2003-2006)

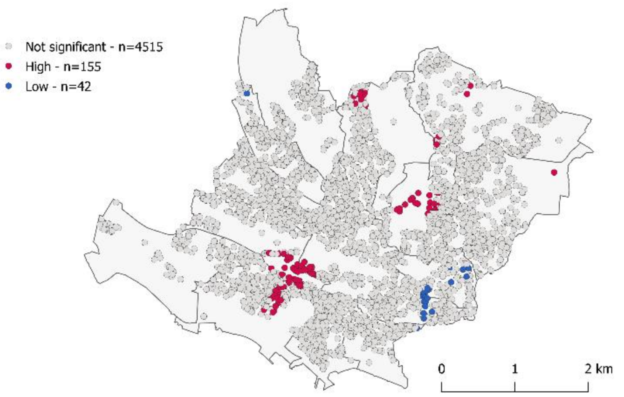

A (2009-2012)

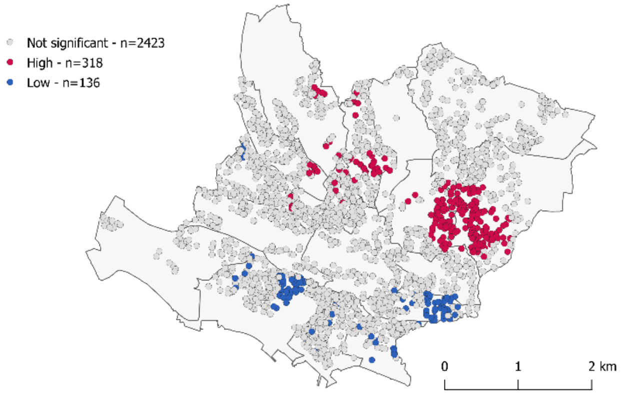

B (2009-2012)

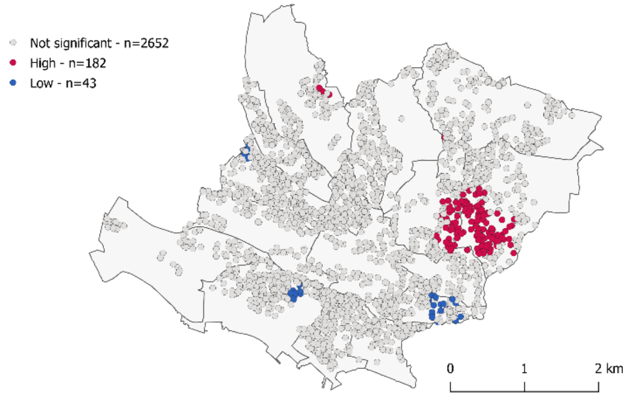

Gi* Getis-Ord for the individuals geo-referenced at their postal address, the CoLaus|PsyCoLaus cohort. White dots show sampling places where the space is neutral (no spatial dependence). Red / Blue dots show individuals with a positive / negative Z score considering a p-value < 0·05 (α=0·05), meaning that high / low values cluster, respectively within a spatial lag of 600 m, are found closer together than expected if the underlying spatial process was random. Pseudo-p 0·05 based on a permutation inference with 999 permutations. The autocorrelation on the mean squared error between predicted probabilities or risk and observed values (ASCVD) can be thought of as clustering of a Brier score, as the Gi* statistic consist of a ratio of the weighted average of the values in the neighboring locations, to the sum

of all values. Model 1 included age, comorbidity, lipid modifying and antihypertensive drug, body mass index, alcohol consumption, living in couple, low education status, Townsend index, Swiss origin, anxiety and major depressive disorder, a polygenic risk score and at follow-up 1 a Mediterranean diet score and daily moderate intensity activity.

Supplementary figure S23. Dot maps of ten-year incident ASCVD unadjusted (A), adjusted using predicted risk with age fixed in a GWR model (B)

A (ten-year after 2003-2006)

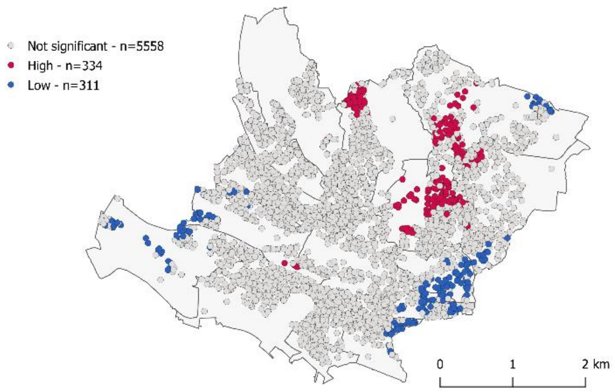

B (ten-year after 2003-2006)

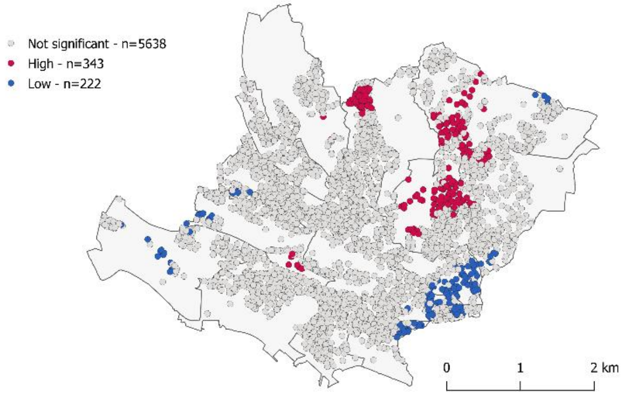

A (ten-year after 2009-2012)

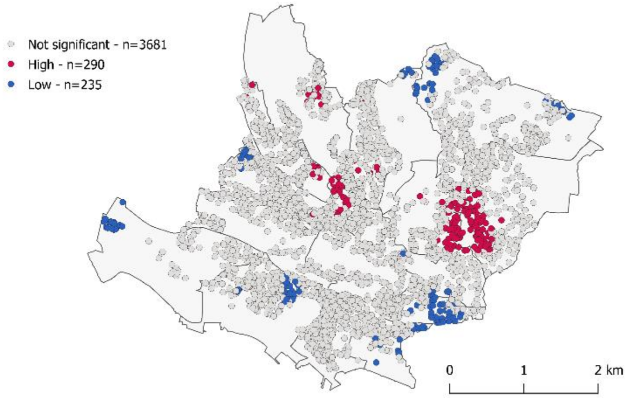

B (ten-year after 2009-2012)

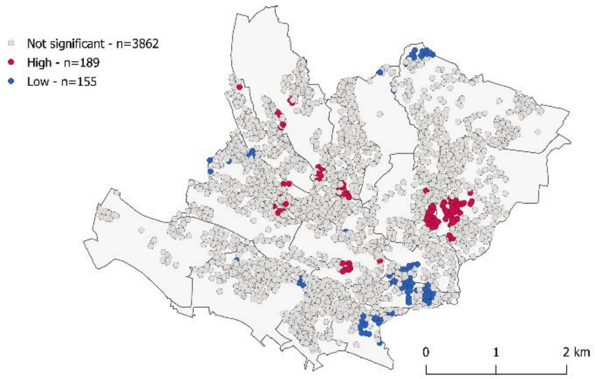

Gi* Getis-Ord for the individuals geo-referenced at their postal address, the CoLaus|PsyCoLaus cohort. White dots show sampling places where the space is neutral (no spatial dependence). Red / Blue dots show individuals with a positive / negative Z score considering a p-value < 0·05 (α=0·05), meaning that high / low values cluster, respectively within a spatial lag of 600 m, are found closer together than expected if the underlying spatial process was random. Pseudo-p 0·05 based on a permutation inference with 999 permutations.
